# Sir2 regulates stability of repetitive domains differentially in the human fungal pathogen *Candida albicans*

**DOI:** 10.1093/nar/gkw594

**Published:** 2016-07-01

**Authors:** Verónica Freire-Benéitez, Sarah Gourlay, Judith Berman, Alessia Buscaino

**Affiliations:** 1 University of Kent, School of Biosciences, Canterbury, Kent CT2 7NJ, UK; 2 Department of Microbiology and Biotechnology, George S. Wise Faculty of Life Sciences, Tel Aviv University, Ramat Aviv, 69978, Israel

## Abstract

DNA repeats, found at the ribosomal DNA locus, telomeres and subtelomeric regions, are unstable sites of eukaryotic genomes. A fine balance between genetic variability and genomic stability tunes plasticity of these chromosomal regions. This tuning mechanism is particularly important for organisms such as microbial pathogens that utilise genome plasticity as a strategy for adaptation. For the first time, we analyse mechanisms promoting genome stability at the *rDNA* locus and subtelomeric regions in the most common human fungal pathogen: *Candida albicans*. In this organism, the histone deacetylase Sir2, the master regulator of heterochromatin, has acquired novel functions in regulating genome stability. Contrary to any other systems analysed, *C. albicans* Sir2 is largely dispensable for repressing recombination at the *rDNA* locus. We demonstrate that recombination at subtelomeric regions is controlled by a novel DNA element, the TLO Recombination Element, TRE, and by Sir2. While the TRE element promotes high levels of recombination, Sir2 represses this recombination rate. Finally, we demonstrate that, in *C. albicans*, mechanisms regulating genome stability are plastic as different environmental stress conditions lead to general genome instability and mask the Sir2-mediated recombination control at subtelomeres. Our data highlight how mechanisms regulating genome stability are rewired in *C. albicans*.

## INTRODUCTION

Repetitive regions clustered at the *rDNA* locus and subtelomeric regions are often the most polymorphic and variable regions of eukaryotes genomes (1–4). Repetitive DNA sequences often undergo homologous recombination which can instigate genomic instability. At these locations, moderate genetic variability is beneficial because it generates the genetic diversity driving evolution and allows adaptation to different environmental niches. However, excessive genome instability is deleterious and an optimum balance between genome integrity and instability is essential for ensuring fitness while permitting adaptation. This is particularly important for microbial pathogens that utilise genome plasticity as a strategy to rapidly and reversibly adapt to different environmental niches. Fungal pathogens are a leading cause of human mortality worldwide, especially in immunocompromised individuals (5). Among those, *Candida albicans*, the principal causal agent of mycotic death, is a highly successful pathogen due in great part to its genome plasticity (6). Natural isolates exhibit a broad spectrum of genetic and genomic variations including single nucleotide polymorphisms (SNPs), short and long range loss of heterozygosity (LOH) events and whole chromosome aneuploidy (7). Environmental stimuli, including exposure to the mammalian host, drug treatment and heat shock, alter the rate and type of chromosomal rearrangements that provide a selective growth advantage in specific environmental conditions (8,9). For example, under standard laboratory growth conditions, most chromosomal rearrangements are LOH events driven by break-induced replication (BIR) where a double stranded DNA break is repaired by invasion of the broken end into a homologous DNA sequence until it reaches the end of the chromosome (8). In contrast, treatment with fluconazole, the most used anti-fungal drug, or 39°C, mimicking host fever, triggers aneuploidy and long range LOH (6). Exposure to hydrogen peroxide (H_2_O_2_), mimicking reactive oxygen species by the host's immune cells, leads to high rates of short range LOH (8).

In many eukaryotes, genome stability at the repetitive *rDNA* locus is ensured by the assembly of a transcriptionally silent chromatin structure, heterochromatin, which suppresses unequal recombination events (1). Heterochromatin is characterised by a specific histone modification pattern controlled by histone modifiers (10,11). For example, heterochromatin in the budding yeast *Saccharomyces cerevisiae* is marked by hypoacetylated nucleosomes. In this system, the histone deacetylase (HDAC) Sir2 deacetylates histone 4 on lysine 16 (H4K16) (10). In other systems, such as the fission yeast *Schizosaccharomyces pombe*, heterochromatin is marked by hypoacetylated nucleosomes that are methylated on lysine 9 of Histone H3 (H3K9me) (12). The histone methyltransferase SuVar3-9 specifically methylates H3K9 (13). The *S. cerevisiae* epigenome is devoid of H3K9 methylation as a SuVar3-9 orthologous is absent in this organism.

A role for heterochromatin in repression of *rDNA* recombination is well established in *S. cerevisiae* where Sir2-dependent hypoacetylated heterochromatin suppress unequal recombination events by repressing non coding transcription and ensuring high levels of cohesion (14–16). The *S. cerevisiae* Monopolin complex, composed of the protein Csm1 and Lrs4, acts in parallel and independently of Sir2 to promote *rDNA* stability by aligning sister chromatids, ensuring silencing, and mediating perinuclear anchoring (17,18). Indeed, deletion of both *S. cerevisiae* Sir2 and Monopolin components results in a dramatic increase of unequal *rDNA* recombination compared to single mutants (17).

Telomeric and subtelomeric regions are also assembled into transcriptionally silent heterochromatin. The role of heterochromatin in controlling genome stability at subtelomeres is still unclear. Indeed, although components of the Sir2-containing protein complex maintain subtelomeric DNA stability (18), mitotic recombination rate at subtelomeric regions is unaltered in cells deleted for Sir2 compared to wild-type (WT) cells (19).

Mechanisms ensuring genome stability at *C. albicans* repetitive elements are unknown. The *C. albicans* genome is composed of 8 diploid chromosomes (20). Similarly to *S. cerevisiae*, the *C. albicans rDNA* locus consists of a tandem array of a ∼12 kb unit repeated 50–200 times on chromosome R. Each unit contains the two highly conserved 35S and the 5S rRNA genes that are separated by two Non-Transcribed Regions (NTS1 and NTS2) (20). The *C. albicans rDNA* locus is highly plastic as the number of *rDNA* units can vary among different *C. albicans* strains (7) with extra-chromosomal plasmids containing several *rDNA* units present in different isolates (21,22).

The 16 *C. albicans* telomeric regions are formed by a terminal element composed of 23 bp tandem repeats and subtelomeric regions containing transposons and subtelomeric genes (1). Among those, the telomere-associated *TLO* genes are a family of 14 closely related paralogues encoding a set of related Med2 subunits for the Mediator transcription regulation complex (23–25). All *TLO* genes are oriented similarly with transcription proceeding toward the centromeres. *TLO* genes can be subdivided in three clades (α, β and γ) based on the presence of indels (25). There are 5 *TLO α*, 1*TLO β* and 7 *TLO* γ genes. These subtelomeric genes are organised in 3 regions: a 5′ region, with homology to *MED2*, that is conserved across all the three clades, a middle region of variable length that contains gene-specific adenosine stretches and a 3′ region that is conserved within a clade but not across clades (25). The presence of a TLO gene family is unique to *C. albicans* as most of the less pathogenic non-albicans species have only one TLO gene. *C. dubliniensis*, the closest relative to *C. albicans*, has two *TLO* genes: an internally located *TLO1* gene and a subtelomeric *TLO2* gene (26). Due to the highly repetitive nature of subtelomeric regions, which precludes detailed analyses by next-generation sequencing, the genetic plasticity of *C. albicans TLO* genes is still poorly understood. However, targeted Sanger sequencing has shown that these regions exhibited hypervariation between clinical isolates (7) and movement of *TLO* genes via recombination occurs at detectable levels within lab-passaged cultures (27).

We have shown that, in *C. albicans*, the repetitive DNA sequences associated with the *rDNA* locus, telomeres and subtelomeric regions, are assembled into hypoacetylated and hypomethylated heterochromatin that lacks H3K9 methylation (28). At these loci, heterochromatin stochastically silences expression of endogenous transcripts as well as of inserted marker genes. The chromatin and transcriptional repressive state associated with heterochromatic regions is dependent on the HDAC Sir2 (28). It is unknown whether Sir2 promotes genome stability of these repetitive regions. In this study, we address this question.

Contrary to any other systems analysed, we find that Sir2 is largely dispensable for repressing recombination at the *rDNA* locus. At this location, the Monopolin complex is the major regulator of recombination.

At subtelomeric regions, we have identified a previously undefined 300 bp DNA sequence, that we named TLO recombination element (TRE), as a site that can promote recombination of a subset of TLO genes. We find that under standard growth conditions, Sir2 represses recombination at the TRE sequence. Under stress conditions, such as treatment with the anti-fungal drug fluconazole or with H_2_O_2_, recombination rates are increased at different genomic loci. This increment in recombination rate is independent of Sir2 and masks the recombination control mediated by Sir2. Our data highlights how the role of the HDAC Sir2 in promoting genome stability has been rewired in *C. albicans*, the most important human fungal pathogen.

## MATERIALS AND METHODS

### Growth conditions

Yeast cells were cultured in rich medium (YPAD) containing extra adenine (0.1 mg/ml) and extra uridine (0.08 mg/ml), complete SC medium (Formedium™) or SC Drop-Out media (Formedium™). When indicated, media were supplemented with 5-fluorotic acid (5-FOA, Melford) at a concentration of 1 mg/ml, Nourseothricin (clonNAT, Melford) at a concentration of 100 μg/ml, Fluconazole (Sigma) at a concentration of 1 μg/ml or 0.5 mM H_2_O_2_. Cells were grown at 30 or 39°C as indicated.

### Yeast strain construction

Strains are listed in Supplementary Table S1. Integration and deletion of genes was performed as previously described (29) using long oligos-mediated PCR for gene deletion and tagging. Oligonucleotides and plasmids used for strain constructions are listed in Supplementary Table S2 and Supplementary Table S3, respectively. Transformation was performed by electroporation (Gene Pulser™, Bio-Rad) using the protocol described in (30). The *URA3* marker gene was used for silencing assay. *HIS1, ARG4* and *SAT1* marker genes were used to delete both copies of *SIR2* and *CSM1* genes. HA tag was used for *SIR2* tagging at the C-terminus. Correct integration events were checked by PCR using primers listed in Supplementary Table S2.

### TRE plasmid construction

pTRE-URA3 was constructed using plasmid pGEMURA3 (29). The TRE (TLO Recombination Element) sequence located at 3′of *TLOα10* was PCR amplified from *C. albicans* genomic DNA using oligos containing the restriction sites SacII (Supplementary Table S2). PCR purified TRE product and pGEMURA3 plasmid were digested with SacII (Promega), ligated and transformed in *DH5-α E. coli* cells. Positive transformants were confirmed by PCR and sequencing with primers listed in Supplementary Table S2.

### Silencing assay in liquid media

Growth analyses were performed using a plate reader (SpectrostarNano, BMG labtech) in 96-well plate format at 30°C. For each silencing assay, 1:100 dilution of an overnight culture was inoculated in a final volume of 95 μl of SC or SC-URA media to reach a concentration of 60 cells/μl. Growth was assessed by measuring *A*_600_, using the following conditions: OD_600 nm_, 616 cycle time, three flashes per well, 700 rpm shaking frequency, orbital shaking mode, 545 s additional shaking time after each cycle 0.5 s post delay, for 32 or 44 h. Graphs represent data from three biological replicates. Error bars: standard deviations of three biological replicates. Data was processed using SpectrostarNano MARS software and Microsoft Excel.

### Fluctuation analysis

Strains were first streaked on –Uri media to ensure the selection of cells carrying the *URA3^+^* marker gene. Fifteen parallel liquid cultures were pre-grown overnight from independent single colonies. Each culture was diluted in YPAD at a concentration of 100 cells/μl and grown for nine generations (18 h). When indicated, media were supplemented with fluconazole (Sigma) at a concentration of 1 μg/ml or 0.5 mM H_2_O_2._ During thermic stress, cultures were grown at 39°C. LOH analyses were performed in three biological replicates. Cells were plated on SC plates containing 1 mg/ml 5-FOA (5-fluorotic acid, Sigma) and on non-selective SC plates and grown at 30°C. Colonies were counted after 2 days of growth and data were analysed using FALCOR (Fluctuation Analysis Calculator) software (31) based on the Lea–Coulson analysis of the median (32). Statistical differences between samples were calculated using Kruskal–Wallis test. Statistical analysis and violin plots were generated using R (http://www.r-project.org/). To quantify the number of colonies able to grow on FOA due to *URA3^+^* silencing, FOA resistant colonies were replica-plated, after fluctuation analysis, on complete SC plates for recovery. Single colonies were then replica-plated on SC plates supplemented with 1 mg/ml 5-FOA and –Uri SC Drop-Out plates. After 24 hour growth single colonies were counted and analysed using Microsoft Excel. To analyse the presence of the SAT1 marker gene after fluctuation analysis, FOA resistant single colonies were replica-plated on complete SC plates for recovery. Single colonies were then replica-plated on YPAD plates containing extra adenine (0.1mg/ml), extra uridine (0.08mg/ml) and Nourseothricin (clonNAT, Melford) at a concentration of 100 μg/ml. After 24 hour colonies were counted and analysed using Microsoft Excel.

### Marker gene loss assay

Strains were first streaked on –Uri media to select for cells carrying the *URA3^+^* marker gene. 15 parallel liquid cultures were pre-grown overnight from independent single colonies. Each culture was diluted in YPAD at a concentration of 100 cells/μl and grown for 9 generations (18 hours). Cells were plated on SC plates containing 1 mg/ml 5-FOA (5-fluorotic acid, Sigma) and on non-selective SC plates and grown at 30°C. To distinguish between silencing and loss of the *URA3* marker gene, FOA resistant single colonies were streaked onto complete SC plates for recovery and then streaked onto –Uri SC Drop-Out plates. Colonies not able to grow on –Uri plates but FOA resistant were counted. Data were analysed using Microsoft Excel. Statistical differences between samples were tested using unpaired t-test using R (http://www.r-project.org/).

### RNA extraction and cDNA synthesis

RNA was extracted from log_2_ exponential cultures (OD600nm = 1.4) using a yeast RNA extraction kit (E.Z.N.A.^®^ Isolation Kit RNA Yeast, Omega Bio-Tek) following the manufacturer′s instructions. RNA quality was checked by electrophoresis under denaturing conditions in 1% agarose, 1X HEPES, 6% Formaldehyde (Sigma). RNA concentration was measured using a NanoDrop ND-1000 Spectrophotometer. cDNA synthesis was performed using iScript™ Reverse Transcription Supermix for RT-qPCR (Bio-Rad) following manufacturer's instructions and a Bio-Rad CFXConnect™ Real-Time System.

### RT-qPCR reactions

Primers used are listed in Supplementary Table S2. RT-qPCR was performed in the presence of SYBR Green (Bio-Rad) on a Bio-Rad CFXConnect^TM^ Real-Time System. Data was analysed with Bio-Rad CFX Manager 3.1 software and Microsoft Excel. Enrichment was calculated over actin. Histograms represent data from three biological replicates. Error bars: standard deviation of three biological replicates generated from 3 independent cultures of the same strain.

### Protein extraction and Western blotting

Yeast extracts were prepared as described (33) using 1 × 10^8^ cells from overnight cultures grown to a final OD600 of 1.5–2. Protein extraction was performed in the presence of 2% SDS (Sigma) and 4 M acetic acid (Fisher) at 90°C. Proteins were separated in 2% SDS (Sigma), 40% acrylamide/bis (Biorad, 161-0148) gels and transfer into PVDF membrane (Biorad) by semi-dry transfer (Biorad, Trans Blot SD, semi-dry transfer cell). Western-blot antibody detection was used using antibodies from Roche Diagnostics Mannheim Germany (Anti-HA, mouse monoclonal primary antibody (12CA5 Roche, 5 mg/ml) at a dilution of 1:1000, and anti-mouse IgG-peroxidase (A4416 Sigma, 0.63 mg/ml) at a dilution of 1:5000, and Clarity™ ECL substrate (Bio-Rad).

### Bioinformatics analysis

*Candida albicans* and *C. dubliensis* TLO genes and flanking sequences were downloaded from the *Candida* Genome Database (34). DNA alignment was performed using Muscle with default setting and visualised using Jalview (35). Motif finder analyses was performed using MEME SUITE using the MEME discovery programme in discriminative mode (36).

### SNP-RFLP analysis

PCR Primers and Restriction Enzymes were chosen according to (37). PCRs were performed in a final volume of 15 μl using Taq DNA polymerase (VWR, 733–1364) using manufacturer's instructions. DNA was extracted from single colonies following NaOH heat extraction. PCR conditions were performed as follow: initial denaturation at 94°C for 7 min, 30 cycles each of denaturation at 94°C for 45 s, annealing at 55°C for 1 min, and extension at 72°C for 1 min, and a final extension at 72°C for 7 min. Each PCR product was digested overnight with the relevant restriction enzyme, AseI for SNP 1, TaqI for SNP 85 and DdeI for SNP135 (37). Enzymatic reactions were performed in a total volume of 15 μl with 1 μl RE, 10× restriction buffer, distilled water, and 5 μl of SNP amplified PCR. 15 μl of the digested PCR product was run on a 3% agarose gel (Melford) along with an undigested control PCR sample. Gels were stained with ethidium bromide and photographed. Genotypes were assigned based on banding patterns for each SNP marker as described in (37).

## RESULTS

### The Monopolin complex, but not Sir2, promotes *rDNA* stability

**Figure 1.**
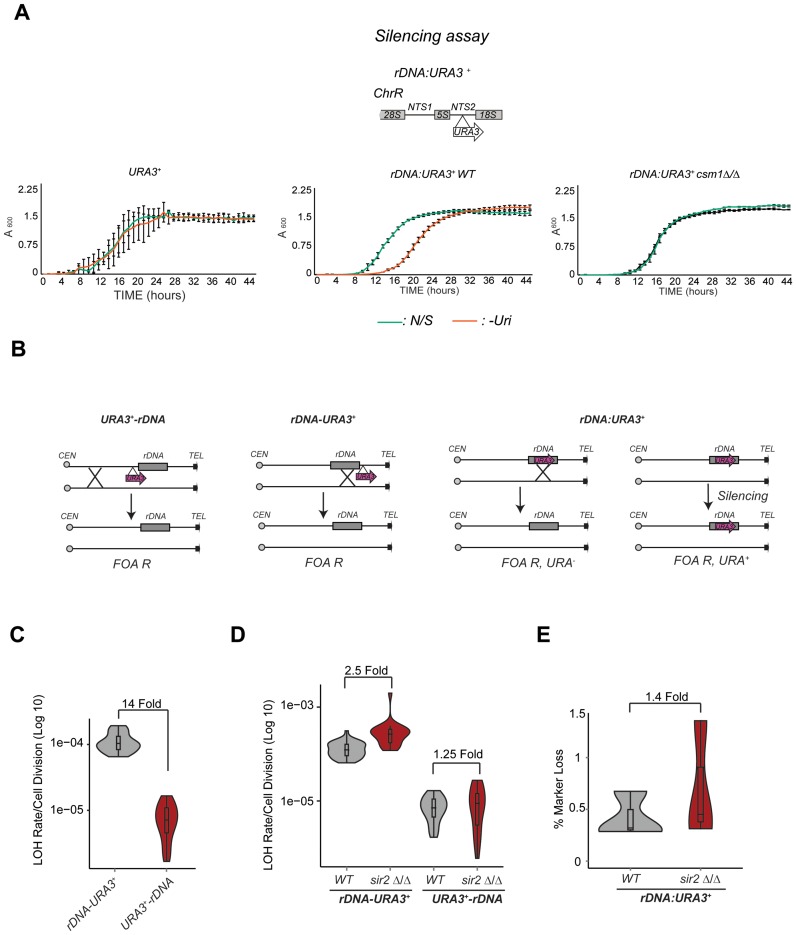
Sir2 does not controls *rDNA* stability. (**A**) *Upper panel:* schematic of *rDNA:URA3^+^* reporter strain. *Bottom panel*: silencing assay of a URA3^+^, *rDNA:URA3^+^* reporter strains in WT and *csm1 Δ/Δ* isolates in non-selective (N/S) and -uridine (-Uri) media. Error bars: standard deviation (SD) of three biological replicates. (**B**) Schematic *URA3^+^-rDNA, rDNA-URA3^+^* and *rDNA:URA3^+^* reporter strains and the mechanism leading to FOA resistance. (**C**) *URA3^+^-rDNA* and *rDNA-URA3^+^* fluctuation analyses. *p*-value calculated with Kruskal–Wallis statistical test is: 2.877 × 10^−09^. (**D**) Fluctuation analyses in *rDNA-URA3* and *URA3^+^-rDNA* in WT, *sir2*Δ/Δ isolates. p-values = 1.302e−07 and 0.665, respectively. (**E**) Percentage (%) marker *URA3^+^* loss with in the *rDNA:URA3* reporter strain in WT and *sir2*Δ/Δ isolates. Violin plots represent all colonies that lost the *URA3+* after fluctuation analysis detected by lack of growth on –Uri media.

In *S. cerevisiae*, it is well established that Sir2 acts in parallel to the Monopolin complex to suppress *rDNA* mitotic recombination (38). We have shown that the *C. albicans* NTS region of the *rDNA* locus is assembled into heterochromatin able to silence an embedded *URA3^+^* marker gene in a silencing reporter strain (*rDNA:URA3^+^*). The HDAC Sir2 maintains this silent state as deletion of Sir2 results in elevated expression of the *URA3^+^* marker gene (28). To assess whether the Monopolin complex contributes to the assembly of silent heterochromatin at the *rDNA* locus, we deleted the *CSM1* gene encoding for the Monopolin component Csm1 in the *rDNA:URA3^+^* reporter strain. Although cells lacking Csm1 grow slower on non-selective (N/S) media compared to WT cells, it is clear that deletion of *CSM1* results in alleviation of *rDNA* silencing (Figure 1A). Therefore, similarly to *S. cerevisiae*, the *C. albicans* Monopolin complex maintains the transcriptionally silenced state associated with the NTS region of the *rDNA* locus.

To assess whether *C. albicans* Sir2 and/or the Monopolin complex control mitotic recombination at the *rDNA* locus, we engineered two *C. albicans* strains where a *URA3^+^* heterozygous marker gene was integrated at centromere-proximal (*URA3^+^-rDNA*) and telomere-proximal side (*rDNA-URA3^+^*) of the *rDNA* locus (Figure 1B). Comparison of the *URA3^+^* loss of heterozygosity between the two strains gives a measure of recombination at the *rDNA* locus because loss of the *URA3^+^* in the *URA3^+^-rDNA* strain detects whole chromosome aneuploidy and/or recombination event upstream of the *rDNA* while loss of the *URA3^+^* marker in the *rDNA-URA3^+^*strain additionally detects recombination events at the *rDNA* locus (Figure 1B). We performed fluctuation analyses where 15 independent cultures were grown for 10 generations before plating on plates containing the *URA3* counter-selective drug FOA. Loss of the *URA3^+^* marker gene was determined by scoring the number of colonies that were able to grow on FOA-containing media compared to N/S media (Figure 1B and C). Importantly, growth of FOA resistant colonies is not a consequence of *URA3^+^* silencing because all the FOA resistant colonies have irreversibly lost the ability to grow on –Uri media (Supplementary Figure S1A and B). Fluctuation analyses in WT strains reveal that the *rDNA* locus is a hotspot of mitotic recombination as mitotic recombination rate of the *rDNA-URA3^+^*gene is 14 fold higher than the mitotic recombination rate of the *URA3^+^-rDNA* gene (Figure 1C). To assess whether *C. albicans* Sir2 controls recombination at the *rDNA* locus as it occurs in *S. cerevisiae*, we deleted both copies of *SIR2* gene at both *rDNA-URA3^+^* and *URA3^+^-rDNA* strains. Contrary to *S. cerevisiae*, we observed only a slight increase in recombination rate in *sir2 Δ/Δ* compared to WT cells (Figure 1D). To assess whether Sir2 represses recombination events within the *rDNA* cluster, we measured, by marker gene loss assay, the number of FOA resistant colonies emerging from a strain with an integrated *URA3^+^*marker gene at the *rDNA* locus (Figure 1B and E). FOA resistant colonies could arise from loss of the *URA3^+^* marker gene following a recombination event or from silencing of the *URA3^+^* marker gene. The first event is irreversible and leads to FOA resistant colonies that are unable to grow on media lacking Uridine (*-Uri)*. In contrast, silencing is reversible and leads to FOA resistant colonies able to grow on *–Uri* plates (Figure 1B). Therefore, to assess the role of Sir2 in controlling rDNA intra recombination, we counted the number of FOA resistant colonies that have irreversibly lost the *URA3^+^* marker gene. As shown in Figure 1E the number of FOA resistant colonies that have lost the *URA3^+^* marker gene following a recombination event is very similar in *sir2 Δ/Δ* versus WT cells (1.4-fold difference) (Figure 1E). Therefore, *C. albicans* Sir2 is not a major contributor of rDNA stability.

**Figure 2.**
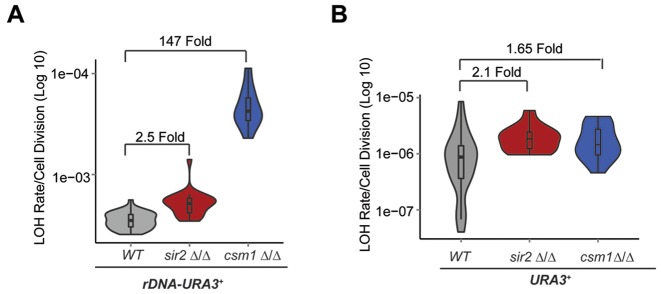
The Monopolin complex, but not Sir2, controls *rDNA* stability. (**A**) *rDNA-URA3* Fluctuation analyses in WT, *sir2*Δ/Δ and *csm1*Δ/Δ cells. *p*-values for WT versus *sir2Δ/Δ* and *csm1Δ/Δ* is respectively: 1.30 × 10 ^−07^ and 2.727 × 10^−06^. (**B**) Fluctuation analyses of a *URA3^+^* heterozygous strain containing a heterozygous *URA3* at its endogenous locus in WT, *sir2*Δ/Δ and *csm1*Δ/Δ cells. p-values = 0.00355 and 0.02623, respectively.

To assess whether the Monopolin complex controls *rDNA* mitotic recombination, we deleted both copies of the *CSM1* gene in the *rDNA-URA3^+^*strain and performed fluctuation analyses. Similarly to *S. cerevisiae, C. albicans* Csm1 represses mitotic recombination rate at the *rDNA* locus as mitotic recombination was 147-fold higher in *csm1 Δ/Δ* strain compared to WT cells (Figure 2A). Importantly, Csm1 specifically represses recombination at the *rDNA* locus as LOH rate of an heterozygous *URA3^+^* gene at its endogenous locus (Chr 3) is similar in WT, *csm1 Δ/Δ* and *sir2 Δ/Δ* cells (2.1 Fold and 1.65 Fold, respectively) (Figure 2B). Therefore, we concluded that *C. albicans* Sir2 does not contribute to *rDNA* stability. Repression of mitotic recombination at this locus is solely dependent on the Monopolin complex that also contributes to transcriptional silencing.

### Loss of heterozygosity is elevated at subtelomeric regions

**Figure 3.**
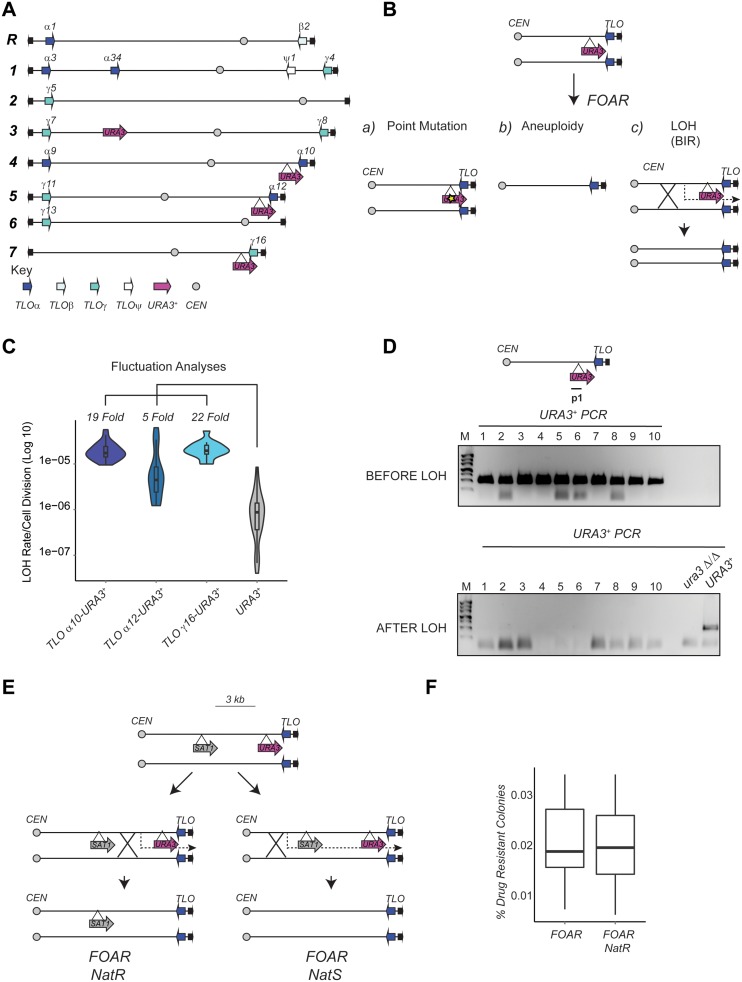
Loss of Heterozygosity is elevated at subtelomeric regions. (**A**) Schematic of *Candida albicans* chromosome organisation. The locations of *TLO* α, β and γ genes are indicated with blue arrows, the locations of the integrated *URA3^+^* marker genes are indicated with magenta arrows. (**B**) Schematic of possible mechanisms leading to FOA resistance. Point mutation: the *URA3^+^* marker is non-functional due to a mutation in the gene sequence. Aneuploidy: the *URA3^+^* marker gene is lost due to a whole-chromosome loss event. Loss of Heterozygosity (LOH): a break-induced recombination (BIR) event leads to loss of the *URA3^+^* marker gene. (**C**) Fluctuation analysis for LOH Rates in *TLOα10-URA3^+^, TLOα12-URA3^+^ and TLOγ16-URA3^+^* compared to the *URA3/ura3Δ* endogenous heterozygous strain (*URA3^+^*). *P*-values, calculated with the Kruskal-Wallis statistical test, are 2.877 × 10^−09^ for *TLOα10-URA3^+^*, 0.003892 for *TLOα12-URA3^+^* and 2.035 × 10^−07^ for *TLOγ16-URA3^+^*. (**D**) *URA3^+^*PCR analyses with primers specific for the *URA3^+^*marker genes was performed with 10 colonies before the Fluctuation analyses (BEFORE LOH) and with 10 FOA resistant colonies (AFTER LOH). A *URA3^+^* and a *ura3 Δ/Δ* strains was included as a positive and negative control. (**E**) Schematics of the *TLO α10-URA3^+^-SAT1* strain. A breaking point between the *SAT1* gene and *URA3^+^* marker gene would produce FoA resistant (FOAR) and NAT resistant (NATR) colonies. A breaking point upstream of both marker genes would produce FOAR colonies that are sensitive to NAT. (**F**) Percentage (%) of drug resistant colonies after fluctuation analyses.

Subtelomeric regions are often the most variable regions of the genome (39–41). Due the complexity of these regions, analysis of subtelomere plasticity is not amenable to genome-wide studies. To analyse *TLO* plasticity in a large population of cells, we set up a system where a heterozygous *URA3^+^* marker gene was integrated at the 3′ end (centromere-proximal) of the *TLO α10, TLO α12* and *TLO γ 6* genes on chromosomes (Chr) 4, 5 and 7, respectively (Figure 3A). Silencing assay demonstrated that at this location the *URA3^+^* marker gene is not silenced as *TLO α10-URA3^+^* and *TLO α 12-URA3^+^* strains grow as well as a *URA3^+^* strain in N/S and –Uri media (Supplementary Figure S2A and B). Fluctuation analyses revealed that FOA resistant colonies appeared more frequently (5–22-fold) when the *URA3^+^* marker gene was inserted centromere-proximal to the three different *TLO* genes than when *URA3^+^* is at its endogenous locus (Figure 3C). This is not the result of reversible silencing as the FOA resistant colonies have irreversibly lost the ability to grow on –Uri media (Supplementary Figure S2C and D). FOA resistant colonies could arise from a point mutation in the *URA3^+^* gene, from whole chromosome loss (with or without regain of the remaining chromosome) or from a telomere-proximal mitotic recombination event (followed by co-segregation of the homologous copies or by BIR) (Figure 3B). The possibility that a point mutation is the major contributor to the appearance of FOA resistance colonies was excluded by PCR analyses with primers specific for the *URA3^+^* gene, as none of the resistant colonies analysed (*n* = 65) retained the *URA3^+^* gene (Figure 3D and Supplementary Figure S3). These results are in agreement with previous observations establishing that in *C. albicans* mutation rate is much lower (∼1000-fold) than the LOH rate (8). Furthermore, these data establish that, similarly to other systems, *C. albicans* subtelomeric genes are highly unstable and associated with high recombination rates. To distinguish between recombination events that were somewhat centromere-proximal or to whole chromosome events, we inserted a second heterozygous marker gene (*SAT1*) 3 kb upstream of the *URA3^+^*marker gene in the same homologous chromosome creating the reporter strain *TLO α10-URA3^+^-SAT1* (Figure 3E). The *SAT1* marker gene confers resistance to the antibiotic nourseothricin (NAT). If whole chromosome aneuploidy was the cause of the *URA3^+^*marker loss, then FOA resistant colonies should also have lost the *SAT1* marker gene and therefore be sensitive to the antibiotic NAT. On the other hand, if the *URA3^+^*marker was lost as a consequence of a mitotic recombination event near the *TLO* gene, FOA resistant colonies should retain the *SAT1* marker gene and therefore be able to grow on a medium containing the antibiotic NAT. As shown in Figure 3F, most of the FOA resistant colonies retained the SAT1 marker gene and therefore were NAT resistant and not NAT sensitive (Figure 3F). These results indicate that the majority of the *URA3^+^* LOH events are due to cross-overs within 3 kb of the *URA3^+^* gene. Thus, *TLO* instability is largely due to mitotic recombination events occurring very close to the *TLO* genes. An important question is why this region is particularly prone to recombination.

### Sir2 suppresses recombination of *TLOα10* and *TLOα12* genes but not *TLOγ16*

**Figure 4.**
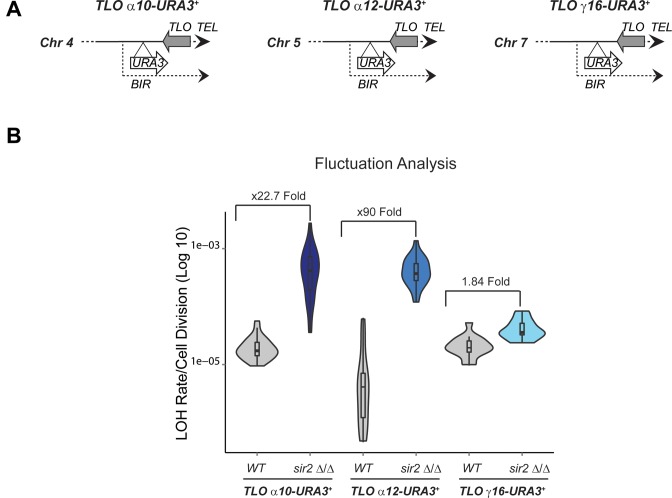
*Sir2* suppresses recombination of *TLOα10* and *TLOα12* genes but not *TLOγ16*. (**A**) Schematic *TLOα10-URA3^+^, TLOα12-URA3^+^ and TLOγ16-URA3^+^* reporter strains. (**B**) for *TLOα10-URA3^+^, TLOα12-URA3^+^ and TLOγ16-URA3^+^* fluctuation analyses in WT and *sir2*Δ/Δ cells. p*-value* for each *TLO* gene in WT versus *sir2Δ/Δ* is respectively: 4.846 × 10^−09^, 2.035 × 10^−07^ and 0.0001475.

We have shown that telomeric regions are assembled into Sir2-dependent heterochromatin (28). To assess whether Sir2 represses mitotic recombination at *TLO* genes, we deleted both copies of the *SIR2* gene in the *TLO* recombination-tester strains on Chr 4, 5 and 7 (*TLOα10-URA3^+^, TLOα12-URA3^+^, TLOγ16-URA3^+^*) (Figure 4A) and performed fluctuation analyses. In WT cells recombination rate associated with these *TLO* genes is similar (Figure 4B). However, LOH rate for *TLOα10-URA3^+^*and *TLOα12-URA3^+^* increased in *sir2 Δ/Δ* compared to WT cells (23 and 90 fold respectively) (Figure 4B). In contrast, *TLOγ16-URA3^+^*recombination rate was increased only marginally, by 1.8-fold, in *sir2 Δ/Δ* cells (Figure 4B). We concluded that Sir2 represses mitotic recombination at *TLOα10* and *TLOα12* but not *TLOγ16*.

### Sir2 represses mitotic recombination at *TLO* genes via a 300 bp TLO recombination element

Recombination occurs in a 3 kb telomeric-distal region of the *TLOα10* and TLOα12 genes and it is dependent on the HDAC Sir2. In contrast, Sir2 does not repress recombination at *TLOγ16* gene. We hypothesised that a *cis*-acting DNA element promotes recombination of *TLOα10* and *TLOα12*, but not of the *TLOγ16*, and that Sir2 acts on this element to repress recombination. To identify this putative control region, we aligned all *TLO* genes and their downstream sequences. This alignment reveals that a 300 bp region downstream of the *TLO* stop codon, that we named TRE, is conserved among all chromosome ends except for the subtelomeric region containing *TLOγ16* gene (Chr 7R) and *TLOβ2* (Chr RR) genes (Supplementary Figure S4). Blast analysis reveals that the TRE element is not present in any other locations in the *C. albicans* genome and it is poorly conserved at the 3′ region of the subtelomeric *C. dubliniensis TLO2* gene (Supplementary Figure S5A). This finding raises the possibility that the TRE element is important for the TLO gene expansion observed in *C. albicans*. Motif finder analysis identifies a 50 nt long motif present in all *TLO* genes except *TLOβ2* and *TLOγ16* (Supplementary Figure S5B). This motif corresponds to the previously identified BTS (Bermuda Triangle Sequence), a site of recombination detected during *C. albicans* evolution experiments (27).

**Figure 5.**
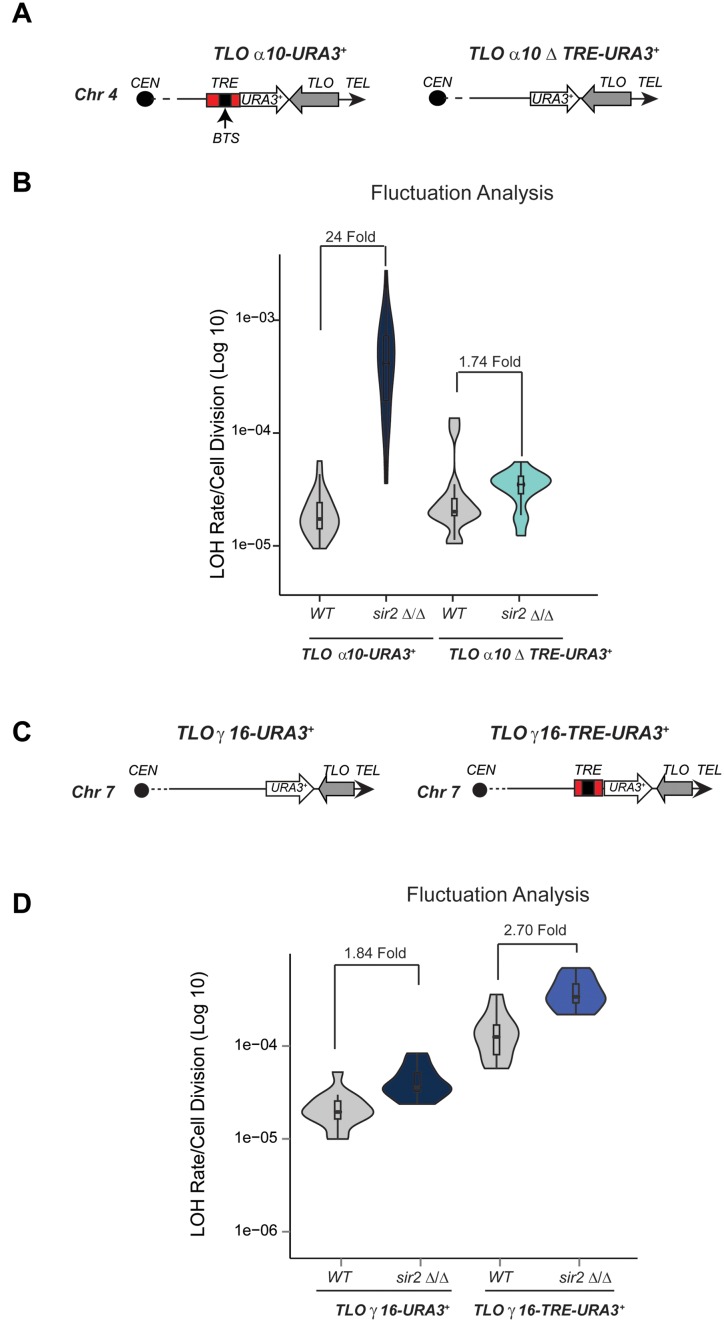
*Sir2* represses mitotic recombination at *TLO* genes via a 300 bp *TLO* Recombination Element. (**A**) Schematic of *TLOα10-URA3^+^* and *TLOα10ΔTRE-URA3^+^* reporter strains. The TRE region and the BTS regions are highlighted. (**B**) *TLO10-URA3^+^, TLOα10 Δ TRE-URA3^+^* fluctuation analysis in WT and *sir2*Δ/Δ cells. (**C**) Schematic of *TLOγ16-URA3^+^, TLOγ16- TRE-URA3^+^*. (**D**) *TLOγ16-URA3^+^, TLOγ16- TRE-URA3^+^* fluctuation analyses in WT and *sir2*Δ/Δ cells. *p*-value = 3.067 × 10^−06^.

To determine whether the TRE element is necessary to promote recombination in the absence of Sir2, we replaced it with a *URA3^+^* marker gene creating the *TLOα10 ΔTRE-URA3^+^* reporter strain lacking the TRE element and containing a heterozygous *URA3^+^* gene in its place (Figure 5A). Fluctuation analyses reveal that recombination rate at *TLOα10-ΔTRE-URA3^+^* did not significantly increase in *sir2 Δ/Δ* compared to WT cells (Figure 5B). Therefore, we concluded that TRE element is necessary to promote recombination and that Sir2 acts via TRE to repress recombination of *TLO* genes that have an adjacent TRE.

To test whether the TRE element is sufficient to induce recombination in the absence of Sir2, we integrated the TRE element together with a *URA3^+^* marker gene downstream of the *TLOγ16* gene, which normally lacks the TRE and does not show Sir2-dependent recombination repression (Figure 5C). Fluctuation analyses revealed that the ectopic TRE partially increases LOH rate (∼6-fold) in WT cells and that deletion of *SIR2* resulted in an additional increase of ∼3-fold at the *TLOγ16* locus (Figure 5D). Taken together our data demonstrate that the TRE element promotes high levels of recombination and that Sir2 represses recombination of *TLO* genes containing the TRE element.

### Stress conditions trigger repeats-associated instability independently of Sir2

**Figure 6.**
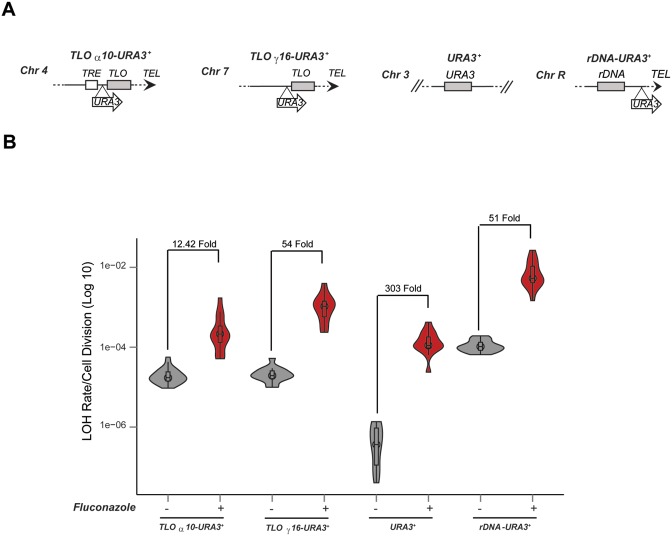
Stress conditions increase LOH associated with all genomic loci tested. (**A**) Schematic of *TLOα10-URA3^+^, TLOγ16-URA3^+^, URA3^+^* and *rDNA-URA3^+^*. (**B**) *TLOα10-URA3^+^, TLOγ16-URA3^+^, URA3^+^* and *rDNA-URA3^+^* fluctuation analysis without (−) and with (+) fluconazole treatment. The calculated *p*-values in the presence and absence of fluconazole for each strain are respectively 2.035 × 10^−07^, 1.125 × 10^−05^, 3.697 × 10^−07^ and 2.035 × 10^−07^.

A range of stress conditions (high temperature, fluconazole treatment and H_2_O_2_ treatment) has been reported to induce *C. albicans* genome instability (8). To assess the effect of stress conditions on the stability of *C. albicans* subtelomeric regions, we asked if treatment with fluconazole, the most common and widely used antifungal drug, affects LOH and/or aneuploidy rate at *TLOα10* (Chr4) and *TLOγ16* (Chr7) (Figure 6A). As a control, we measured LOH rate at the *URA3^+^* endogenous locus (Chr3) and the *rDNA* locus (ChrR) (Figure 6A). Consistent with previous results (8), fluctuation analyses showed that fluconazole treatment results in a dramatic increase of LOH rates at all loci tested indicating that fluconazole leads to general genome instability including subtelomeric regions (Figure 6B). SNP-RFLP analysis of FOA Resistant colonies reveals that fluconazole treatment does not increase whole chromosome aneuploidy of two different chromosomes (Supplementary Figure S6). To assess whether, fluconazole increases long LOH tracts, we performed fluctuation analyses with and without fluconazole in the reporter strain *TLO α10-URA3^+^-SAT1* containing the SAT1 marker gene 3 Kb upstream of the *URA3^+^*marker gene in the same homologous chromosome (Supplementary Figure S7A). Fluconazole treatment does not increase long LOH tracts as all the FOA resistant colonies were also resistant to the antibiotic NAT and therefore loss of the *URA3^+^* marker gene is due to recombination in proximity of telomeres (Supplementary Figure S7B).

**Figure 7.**
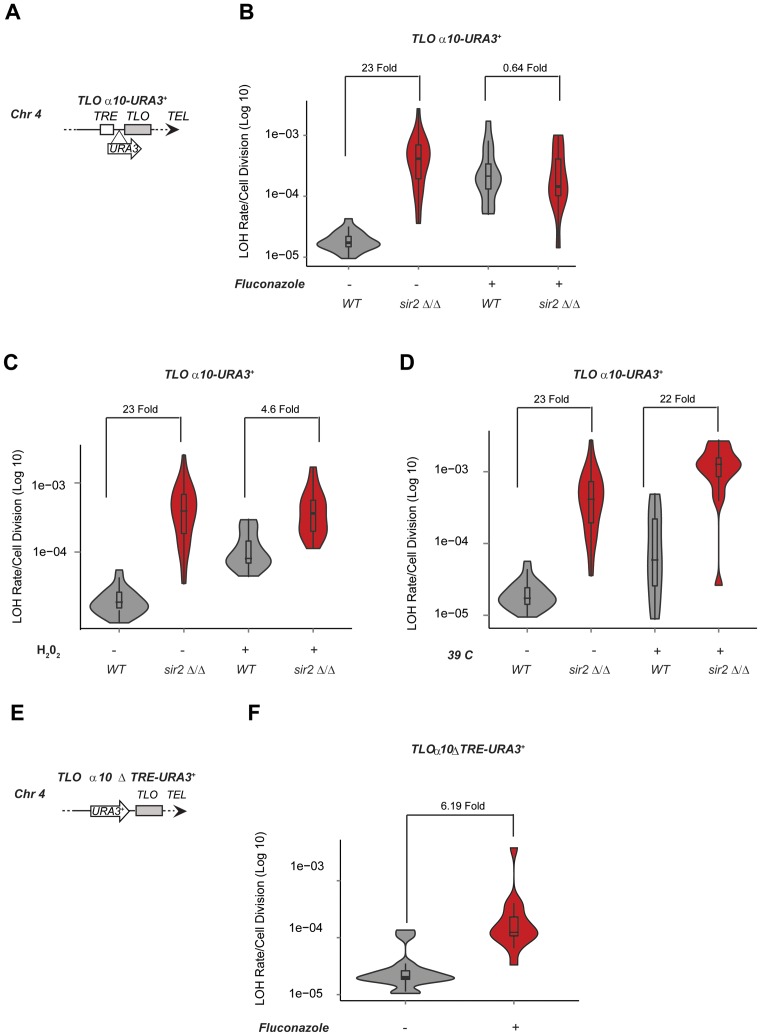
Instability of *TLO* genes triggered by stress conditions masks the action of *Sir2*. (**A**) Schematic of *TLOα10-URA3^+^*. (**B**) *TLOα10-URA3^+^*fluctuation analysis in WT and *sir2*Δ/Δ cells without (−) and with (+) fluconazole treatment. *p*-values = 4.846 × 10^−09^ and 0.6591, respectively. (**C**) Fluctuation analysis for LOH Rates in *TLOα10-URA3^+^* in WT and *sir2*Δ/Δ cells without (−) and with (+) H_2_O_2_ treatment. *p*-values = 4.846 × 10^−09^ and 0.0001475, respectively. (**D**) *TLOα10-URA3^+^* in WT and *sir2*Δ/Δ cells fluctuation analysis at 30°C or 39°C. *p*-values = 4.846 × 10^−09^ and 3.065 × 10^−05^, respectively. (**E**) Schematic of *TLOα10 Δ TRE-URA3^+^*. (**F**) *TLOα10 Δ TRE-URA3^+^* fluctuation analysis without (−) and with (+) fluconazole treatment. *p*-values = 3.065 × 10^−05^.

To assess whether fluconazole treatment impacts on the Sir2-mediated control of recombination, we measured LOH rate of the TRE-containing subtelomere gene *TLO α10* in WT and *sir2 Δ/Δ* cells. While in untreated cells, LOH rate dramatically increases in *sir2 Δ/Δ* compared to WT cells (23-fold) (Figure 7B), in the presence of fluconazole, recombination rate only slightly increases in *sir2 Δ/Δ* compared to WT cells (0.64-fold, Figure 7B). Although less dramatic, H_2_O_2_ treatment leads to similar results. H_2_O_2_ treatment leads to an increase in LOH rate without affecting whole chromosome aneuploidy or long LOH tracts (Figure 7C and Supplementary Figure S8). In addition, following treatment with H_2_O_2_ the increase in LOH rate at *TLOα10-URA3^+^* is much smaller in *sir2 Δ/Δ* cells compared to WT cells (from 23-fold to 4.6-fold) (Figure 7C). Not all stress conditions have the same effect as growing *C. albicans* cells at high temperature (39°C), a temperature mimicking fever in the host, does not abolish the Sir2-mediated recombination control or affects whole chromosome aneuploidy or long LOH tracts (Figure 7D and Supplementary Figure S8). Therefore, the high genome instability instigated by stress conditions (fluconazole and H2O2) masks the recombination control mediated by Sir2. Importantly, fluconazole increases LOH rate independently of the TRE element, as recombination rate at *TLOα10-ΔTRE-URA3^+^*, a reporter strain lacking the TRE element, was still higher following fluconazole treatment (Figure 7E and F). Given that fluconazole does not affect Sir2 RNA and protein level (Supplementary Figure S9), we suggest that stress conditions increase recombination rates independently of Sir2.

## DISCUSSION

This study highlights how the HDAC Sir2 has acquired novel roles in the regulation of repeats-associated genome stability in *C. albicans*, the most common human fungal pathogen.

### The Monopolin complex, but not Sir2, promotes rDNA stability

**Figure 8.**
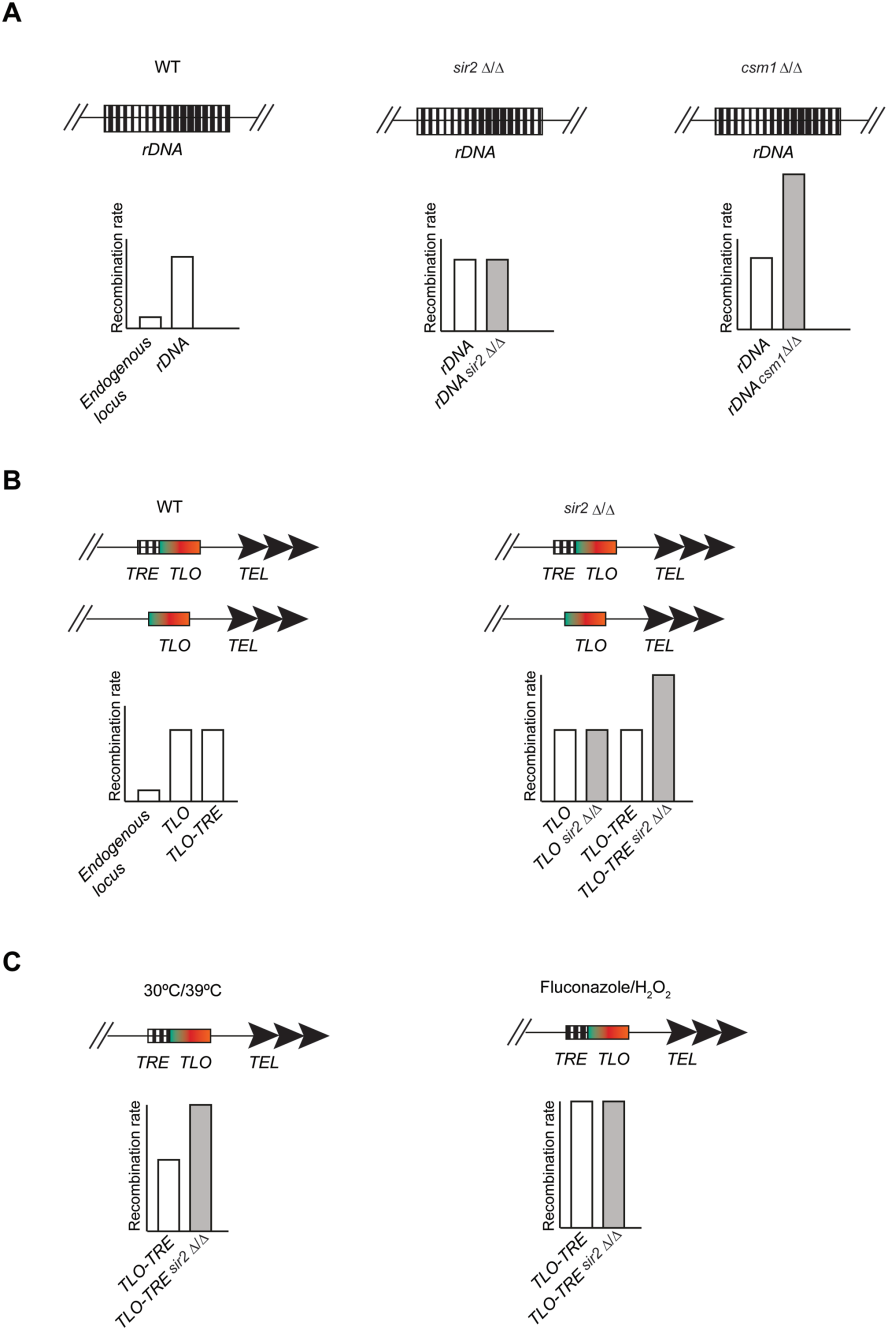
Regulation of genome plasticity in *C. albicans*. (**A**) Schematic model to represent recombination control at the *rDNA* locus in WT, *sir2Δ/Δ* and *csm1Δ/Δ* cells. (**B**) Schematic model to represent recombination control at subtelomeric regions in WT and *sir2Δ/Δ* cells. (**C**) Schematic model to represent recombination rates in control or under stress conditions (30/39°C, fluconazole and H_2_O_2_ treatment) in WT, *sir2Δ/Δ* cells.

In most eukaryotes, the *rDNA* locus is very plastic: the number of *rDNA* units changes in response to nutrients availability (42). However, excessive plasticity is deleterious and regulatory mechanisms have been evolved to ensure *rDNA* integrity. The regulatory network promoting *rDNA* stability is well established in *S. cerevisiae* where the HDAC Sir2 and the Monopolin complex act in parallel to protect *rDNA* repeats integrity (43,44). Here, we demonstrate that *C. albicans* Sir2 has lost the ability to promote *rDNA* stability. In *C. albicans, rDNA* stability is ensured only by the Monopolin complex that also contributes to transcriptional repression (Figure 8A). This observation is surprising and in striking contrast with all the other organisms analysed to date. We propose that heterochromatin assembled at the *rDNA* locus has lost the ability to repress mitotic recombination in order to facilitate karyotype diversity. Indeed, in several *C. albicans* clinical isolates the chromosomal regions distal to the *rDNA* locus are largely homozygous (12), presumably due to a high recombination rate at the *rDNA* array. This strategy could be particularly important for *C. albicans* because it lacks a meiotic cycle and therefore must generate genetic diversity through mitotic events (13,14).

### Recombination control of *C. albicans* subtelomeres

Here, we have analysed mechanisms governing genome stability at *C. albicans* subtelomeres (Figure 8). We found that all subtelomeres are unstable regions of the *C. albicans* genome as recombination rate associated with these regions is much higher than recombination rate associated with an internal not-repetitive region (Figure 8B). High genomic instability is associated with subtelomeres in many organisms and it is thought to play a key role in adaptation and evolution (2). This could be a critical regulatory mechanism in *C. albicans*. This is because *TLO* genes encode proteins with similarity to Med2, a component of the Mediator complex that regulates transcription by RNA polymerase II (45). Recombination between non-allelic *TLO* genes has the potential to introduce primary sequence changes into Tlo proteins encoded by the recombined ORFs. Since the resulting Tlo proteins all have different primary sequences (25), and since mediator complexes include only one Med2 subunit (23), changes in the identity and/or stoichiometry of the *TLO* genes is expected to alter the composition of the mediator complex driving changes in global transcriptional patterns. Consistent with this hypothesis, strains with different *TLO* organisation have altered fitness levels (27).

In many organisms, subtelomeric regions are assembled into transcriptionally silent heterochromatin that stochastically silences gene expression of associated genes (46). In previous work we found that Sir2-dependent heterochromatin assembles over *C. albicans* telomeric regions, where it stochastically represses expression of nearby genes, including *TLO* genes (28,47). In this study, we found that Sir2 controls the recombination dynamics of *C. albicans* subtelomeric regions via TRE, a novel recombination control element located at the 3′ region of a subset of TLO genes. Our data demonstrate that the TRE element has the potential to mediate high levels of recombination and that Sir2 tempers recombination at all *TLO* genes that have an adjacent TRE (Figure 8B). Mechanisms underlying the TRE-recombination control are still unknown. However, it is possible that, similarly to what has been observed at the *S. cerevisiae rDNA* locus, the TRE element could lead to high level of recombination via inducing a replication fork stress and the Sir2 could promote genome stability by repressing non-coding transcription ensuring high levels of cohesion (14–16).

Finally, we found that specific stress conditions, such as Fluconazole and H202 treatment, increase genome instability across the *C. albicans* genome (Figure 8C). Mechanisms that increase genome instability in specific stresses (e.g. fluconazole and H_2_O_2_) operate independently of Sir2 but they can mask the Sir2-dependent recombination control. This is likely because these reagents directly drive chromosome missegregation and/or DNA breaks and Sir2 is not involved in the effect of either fluconazole or H_2_O_2_ on DNA integrity.

In summary, we show that, while *C. albicans* Sir2 does not promote *rDNA* stability, Sir2 ensures stability of subtelomeric genes via the *cis*-acting DNA element TRE. The contribution of Sir2-dependent recombination is independent of mechanisms triggering genomic instability in fluconazole or H_2_O_2_, but appears to be temperature sensitive (Figure 8). This study highlights another layer of complexity in the regulation of DNA repeats plasticity.

## Supplementary Material

SUPPLEMENTARY DATA
