# Supplementary material for: Sir2 regulates stability of repetitive domains differentially in the human fungal pathogen Candida albicans
Source: Nucleic Acids Res. 2016 Jul 1;44(19):9166–79. doi: 10.1093/nar/gkw594 (PMC5100595; doi:10.1093/nar/gkw594)
Supplement: SUPPLEMENTARY DATA [file supp_44_19_9166__index.html]

Sir2 regulates stability of repetitive domains differentially in the human fungal pathogen Candida albicans — Sir2 regulates stability of repetitive domains differentially in the human fungal pathogen Candida albicans — SUPPLEMENTARY DATA 

# Sir2 regulates stability of repetitive domains differentially in the human fungal pathogen *Candida albicans*

## SUPPLEMENTARY DATA

- SUPPLEMENTARY DATA
