# Supplementary material for: Sir2 regulates stability of repetitive domains differentially in the human fungal pathogen Candida albicans
Source: Nucleic Acids Res. 2016 Jul 1;44(19):9166–79. doi: 10.1093/nar/gkw594 (PMC5100595; doi:10.1093/nar/gkw594)
Supplement: SUPPLEMENTARY DATA [file supp_gkw594_nar-01588-v-2016-File010.pdf]

Supporting Information

Sir2 regulates stability of repetitive domains differentially in the human fungal pathogen *Candida albicans*

Verónica Freire-Benéitez[1], Sarah Gourlay[1], Judith Berman[2] and Alessia Buscaino[1]

[1] University of Kent, School of Biosciences Canterbury Kent, CT2 7NJ. UK

[2] Department of Microbiology and Biotechnology, George S. Wise Faculty of Life Sciences, Tel Aviv University, Ramat Aviv, 69978, Israel

Content:

Supplementary Fig S1 and S9

Supplementary Table S1 to S3

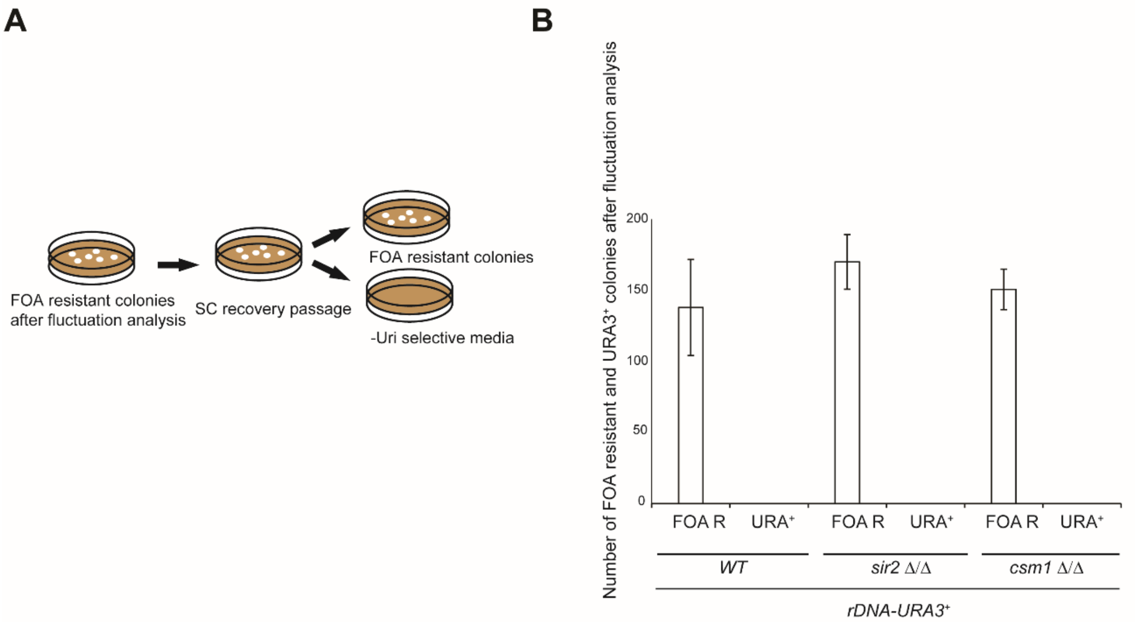

Supplementary figure 1.

(A) Schematic of experimental procedure to detect silencing. Following fluctuation analysis, FOA resistant single colonies were streaked on SC plates and grown for 24 hour. The day after, single colonies were streaked on FOA selective plates and –Uri plates. **(B)** Histogram shows the number of FOA resistant ($URA3^-$) and $URA3^+$ colonies following fluctuation analyses of rDNA-*URA3*$^+$ strain in WT, *sir2 Δ/Δ* and *csm1 Δ/Δ* strain. Error bars: Standard Deviation of three biological replicates.

Freire-Benéitez et al_S2

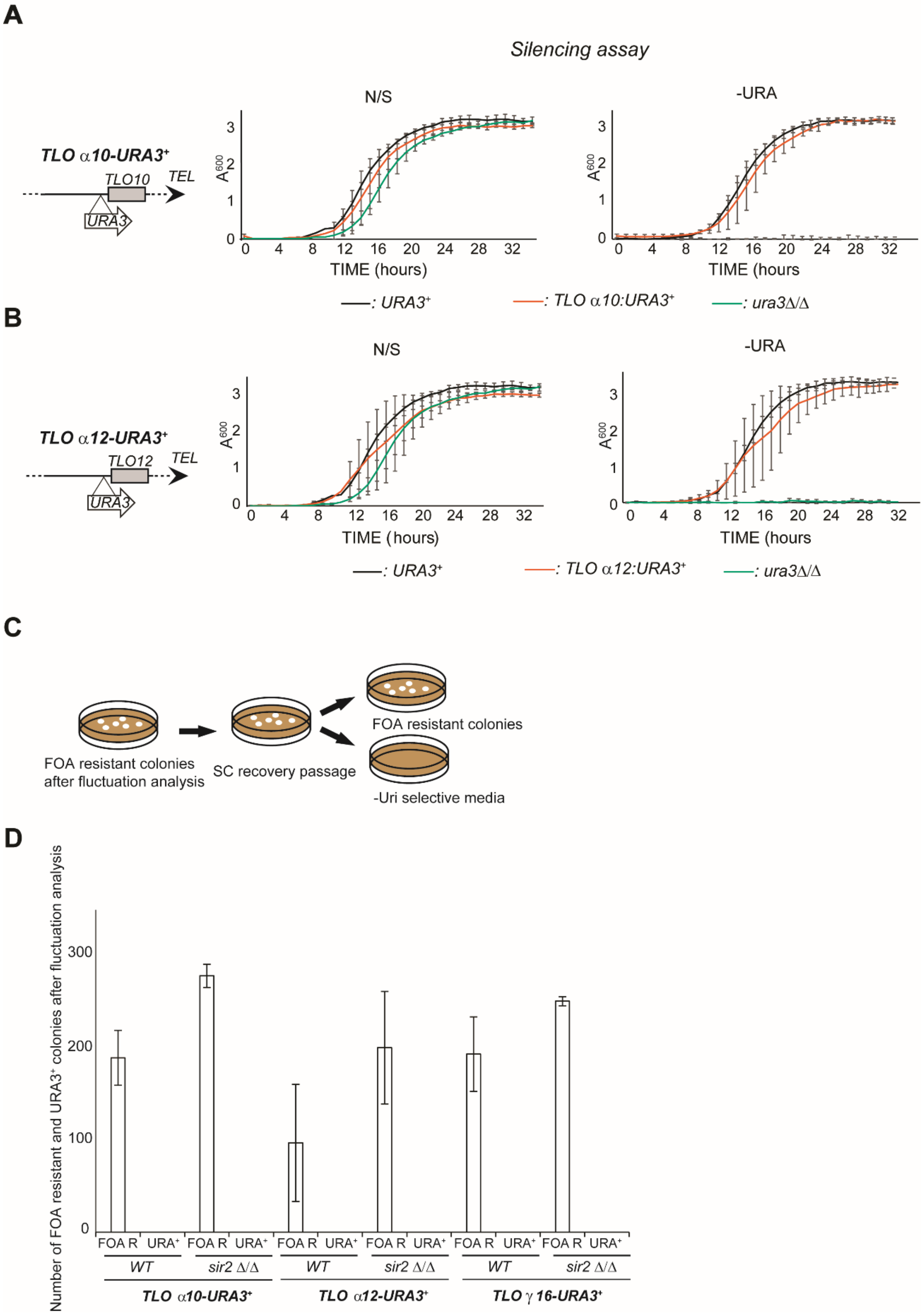

Supplementary figure 2.

(A) Left panel: Schematic of *TLOα10-URA3*$^{+}$ strain. Right panel: silencing assay of the *TLOα10-URA3*$^{+}$ strain. Ura+ (URA3/URA3) and Ura- (ura3Δ/ ura3Δ) strains were included as controls. **(B)** Left panel: Schematic of *TLOα12-URA3*$^{+}$ strain. Right panel: silencing assay of the *TLOα12-URA3*$^{+}$ strain. *URA3*$^{+}$ (URA3/URA3) and *ura*$^{-}$ (ura3Δ/ ura3Δ) strains were included as controls. Error bars in each panel: Standard deviation (SD) of three biological replicates. **(C)** Schematic of experimental procedure to detect silencing following fluctuation analysis. **(D)** Histogram shows the number of FOA resistant (URA3^{-}) and URA3^{+} colonies following fluctuation analyses of *TLOα10-URA3*$^{+}$*, TLOα12-URA3*$^{+}$ and *TLOϒ16-URA3*$^{+}$ strains in WT and *sir2 Δ/Δ* strains. Error bars: Standard Deviation of three biological replicates.

Freire-Benéitez et al_S3

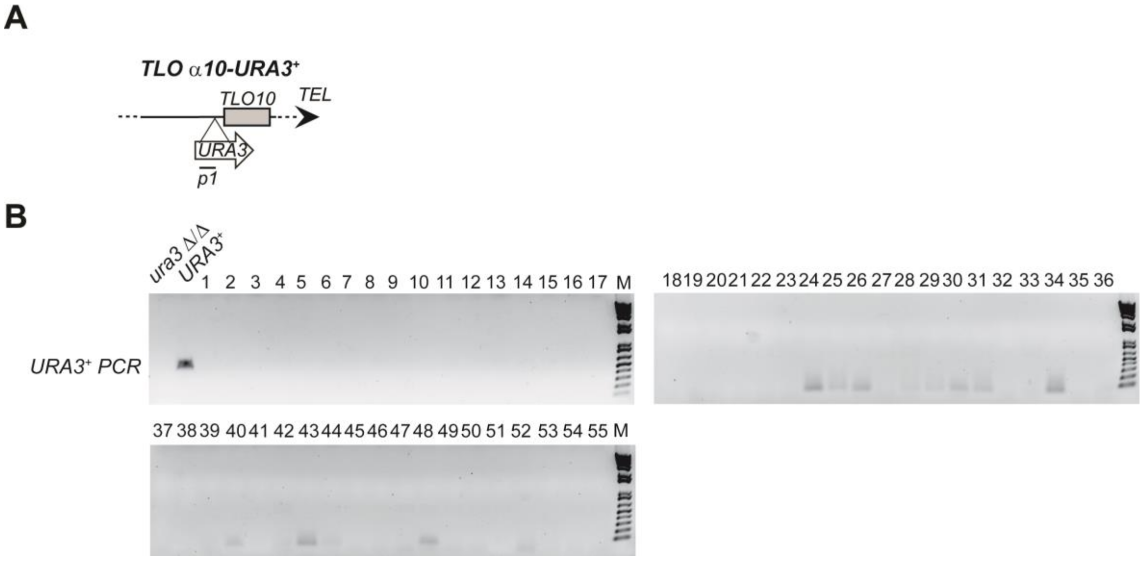

Supplementary figure 3.

(A) Upper panel: Schematic of *TLOα10-URA3*$^{+}$ strain **(B)** *URA3*$^{+}$ PCR analyses with primers specific for the *URA3*$^{+}$ marker gene was performed with 55 FOA resistant colonies (after fluctuation analysis). A *URA3*$^{+}$ and a *ura3 Δ/Δ* strains were included as a positive and negative control.

Freire-Benéitez et al_Fig S4

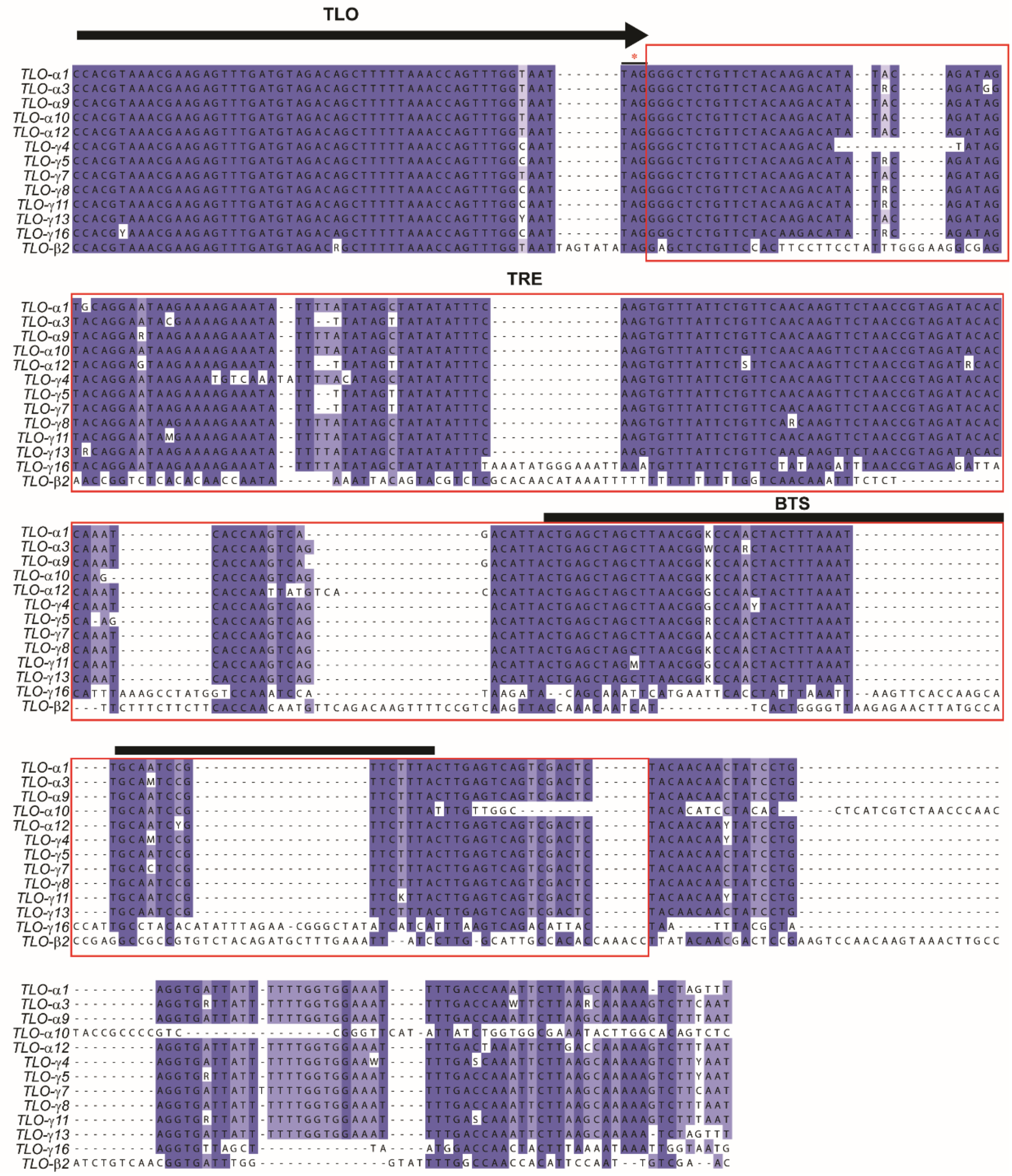

Supplementary figure 4.

Sequence alignment of *TLO* genes and downstream sequences. A red square highlights the 300bp TRE element and a black solid line the 50 bp BTS sequence.

Freire-Benéitez et al_Fig S5

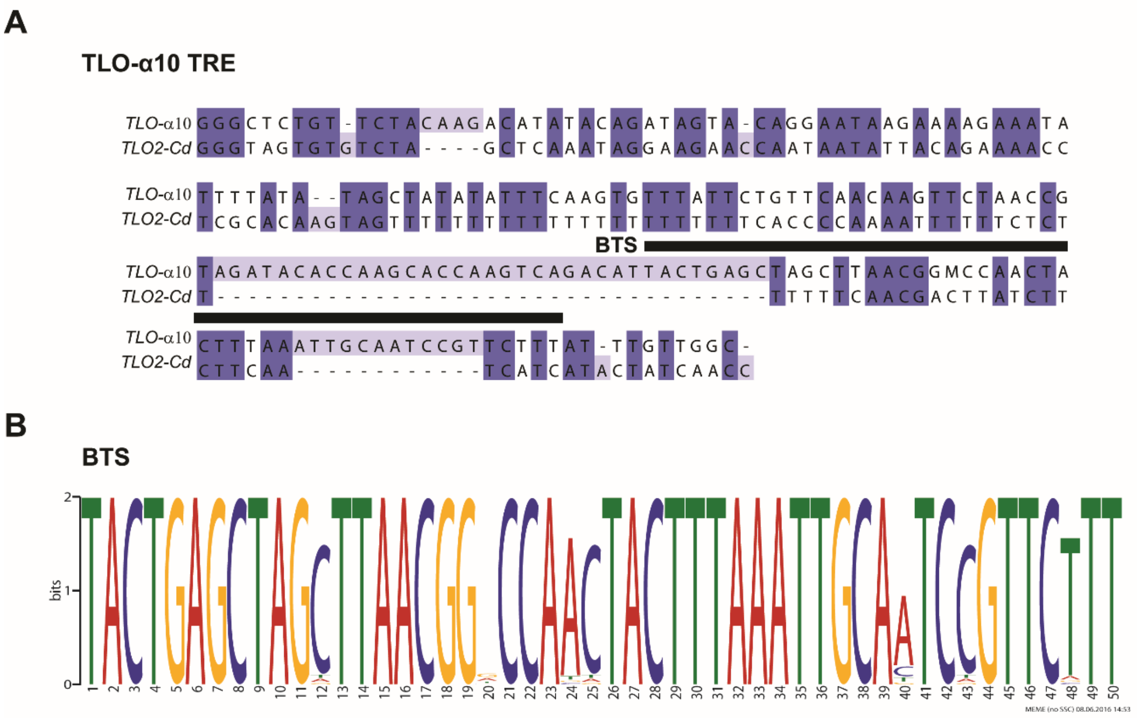

Supplementary figure 5.

(A) Sequence alignment of subtelomeric C.albicans TLOα10 gene and subtelomeric C.dublinensis TLO2. A black solid line highlights the 50 bp BTS sequence. **(B)** De novo motif analysis using MEME on the TRE element identified this highly significant 50 nt sequence (E-value=1.5e−184) corresponding to the BTS sequence.

Freire-Benéitez et al_Fig S6

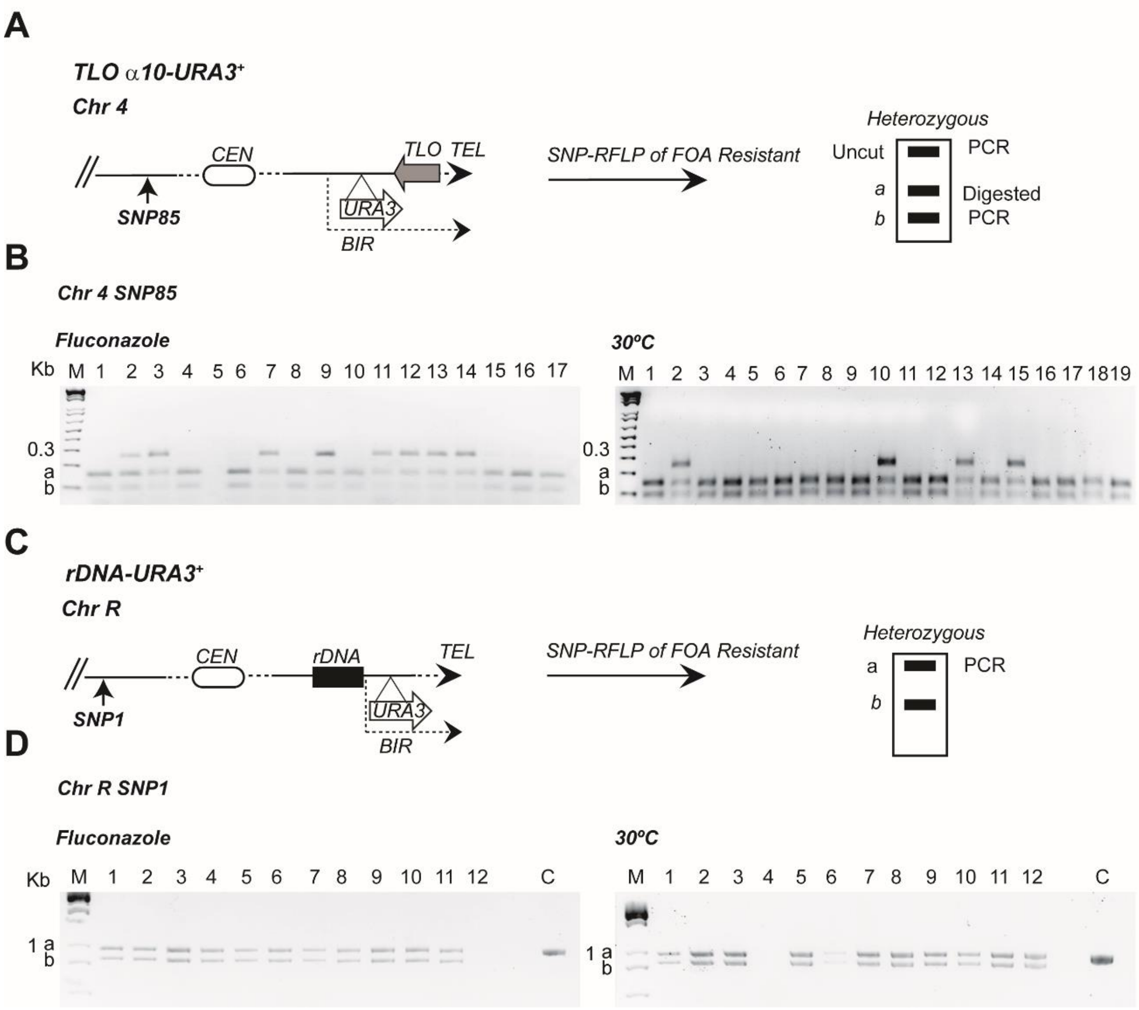

Supplementary figure 6

(A) Schematic of *TLOα10-URA3*+ strain and SNP85 located on the left arm of chromosome 4 and predicted digestion pattern after SNP-RFLP analysis. A different digestion pattern between the two different homologues chromosome (a and b) allows to detect homozygosity or heterozygosity **(B)** Following fluctuation analyses in the presence of absence of 1 µg/ml fluconazole, PCR analyses were performed on 17-19 FOA resistant following by TaqI enzymatic digestion. The digested product was run into a 1.5 % agarose gel. M: 100 bp ladder, the two homologues chromosome (a and b) are indicated **(C)** Schematic of *TLOα12-URA3*+ strain and SNP1 located on the left arm of chromosome R and predicted digestion pattern after SNP-RFLP analysis. A different digestion pattern between the two different homologues chromosome (a and b) allows to detect homozygosity or heterozygosity **(D)** Following fluctuation analyses in the presence of absence of 1 µg/ml fluconazole, PCR analyses were performed on 12 FOA

resistant colonies, the PCR product was digested with AseI. The digested product was run into a 1.5 % agarose gel. A non-digested SNP 1 PCR product was included as a control. M: 100 bp ladder, the two homologues chromosome (a and b) are indicated

Freire-Benéitez et al_Fig S7

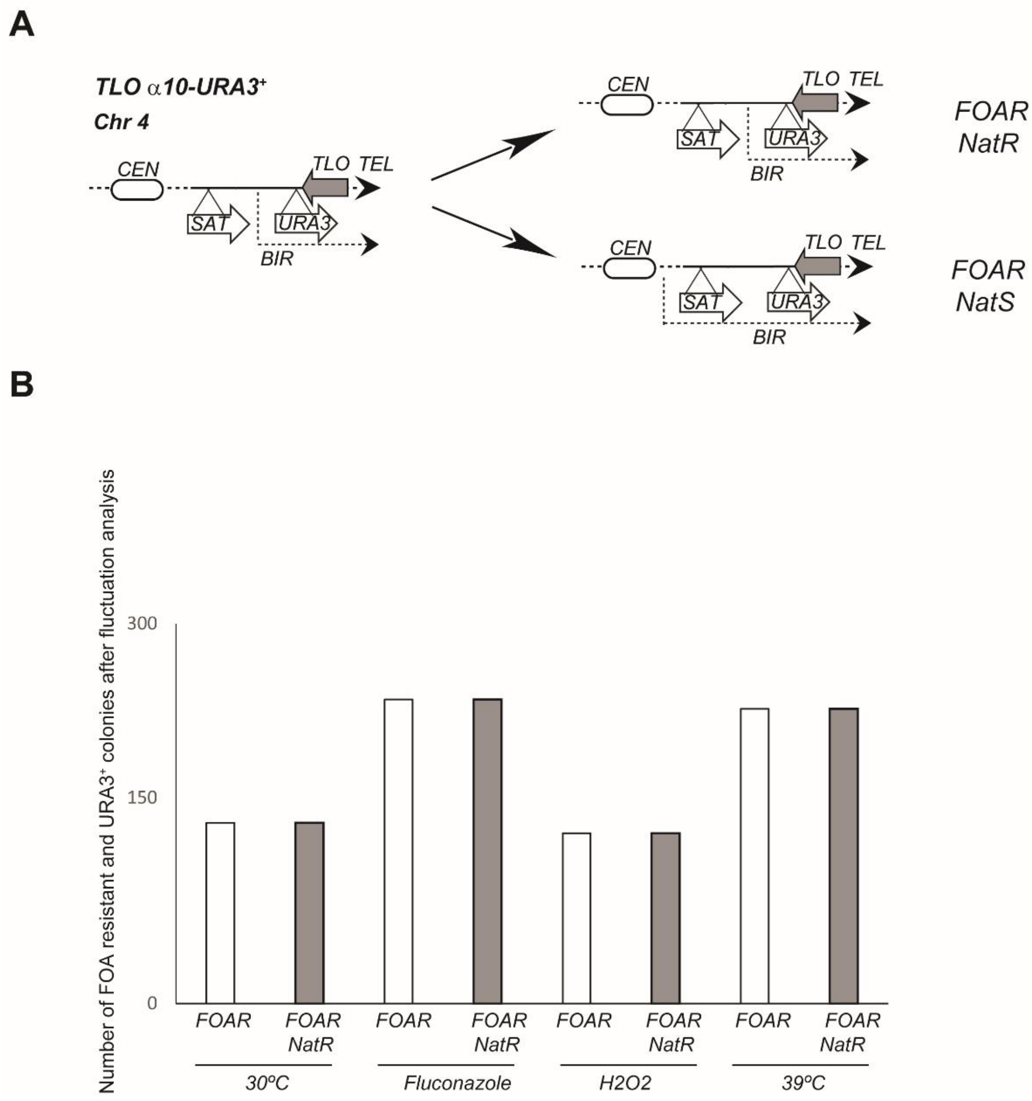

Supplementary figure 7

(A) Schematics of the *TLO α10-URA3*+*-SAT1* strain to confirm LOH range at *TLO α10* locus in the presence of stress conditions. A breaking point between the *SAT1* gene and *URA3*+ marker gene would produce FOA resistant (FoAR) and NAT resistant (NATR) colonies. A breaking point upstream of both marker genes would produce FOAR colonies that are sensitive to NAT **(B)** Schematic of experimental procedure to test whether, following fluctuation analyses, colonies that have lost the *URA3*+ marker gene (FOAR) retain the SAT1 marker gene and, therefore, are NAT resistant. **(E)** Histograms show that most of the FOA resistant colonies analysed after fluctuation analysis in all stress conditions are resistant to the antibiotic NAT.

Freire-Benéitez et al_Fig S8

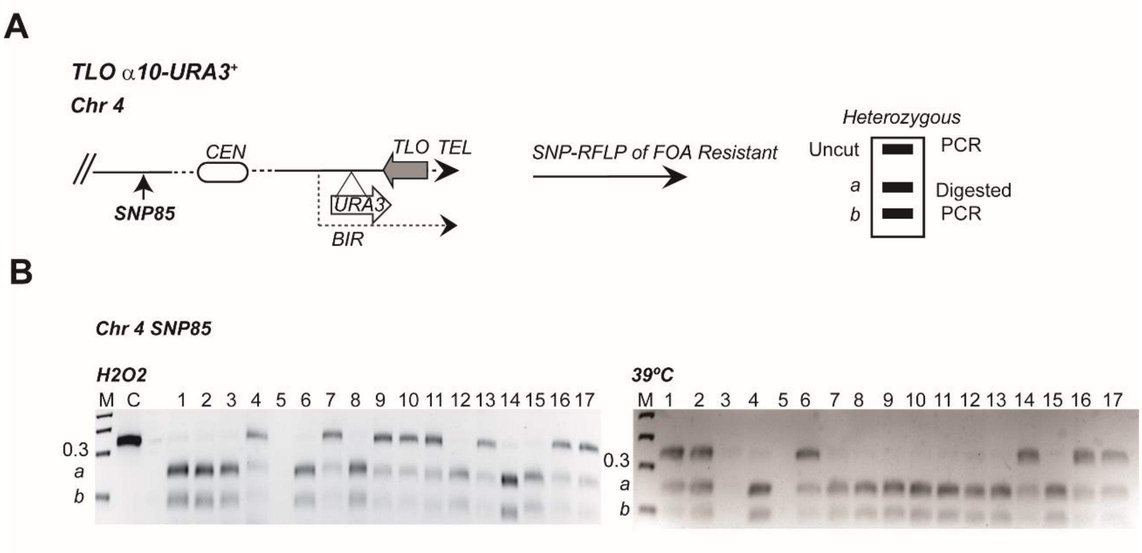

Supplementary figure 8

(A) Schematic of *TLOα10-URA3*+ strain and SNP85 located on the left arm of chromosome 4 and and predicted digestion pattern after SNP-RFLP analysis. A different digestion pattern between the two different homologues chromosome (a and b) allows to detect homozygosity or heterozygosity **(B)** SNP 85 (Forche) PCR analyses coupled with TaqI enzymatic digestion were performed with 17 FOA resistant colonies after LOH in the presence of 0.5mM H_2O_2 and at 39ºC.

Following fluctuation analyses, SNP 85 PCR analyses coupled with TaqI enzymatic digestion were performed on 17 resistant colonies in the presence and absence of 1 µg/ml fluconazole and run into a 1.5 % agarose gel. M: 100 bp ladder, the two homologues chromosome (a and b) are indicated

Freire-Benéitez et al_Fig S9

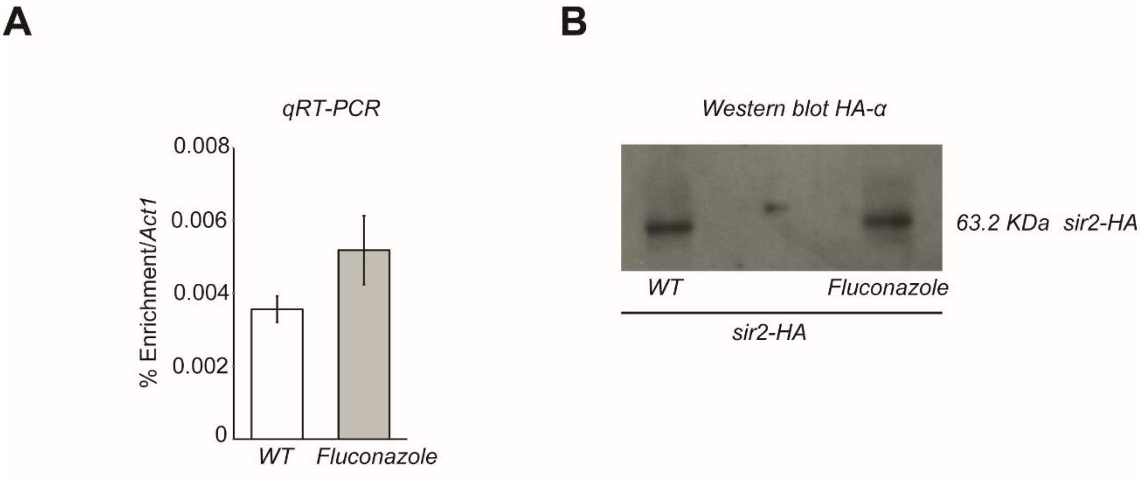

Supplementary figure 9

(A) qRT-PCR analyses to measure *SIR2* transcript levels relative to actin transcript levels (*ACT1*) at 30°C in the presence and absence of fluconazole. Error bars: SD of three biological replicates. **(B)** Western blot to measure Sir2 protein levels in the presence and absence of fluconazole.

Supplementary table 1. *C.albicans* strains used in this study.

Strain number	Description	Genotype
Bu_54	SN152. *URA3 heterozygous* (1)	*MTL a/alpha ura3Δ-iro1Δ::imm434/URA3-IRO1 his1Δ/his1Δ arg4Δ/arg4Δ leu2Δ/leu2Δ*
Bu_95	*rDNA:URA3*$^{+}$ (2)	rDNA::*URA3 ura3Δ::λimm434/ura3Δimm434 his1::hisG/his1::hisG arg4::hisG/arg4::hisG*
Bu_97	*URA3-rDNA*	*URA3-rDNA ura3Δ::λimm434/ura3Δimm434 his1::hisG/his1::hisG arg4::hisG/arg4::hisG*
Bu_102	*rDNA:URA3*$^{+}$ *sir2Δ/Δ* (2)	rDNA *::URA3 ura3Δ::λimm434/ura3Δimm434 his1::hisG/his1::hisG arg4::hisG/arg4::hisG sir2Δ::HIS1/sir2Δ::ARG4*
Bu_117	*URA3-rDNA sir2Δ/Δ*	*URA3-rDNA ura3Δ::λimm434/ura3Δimm434 his1::hisG/his1::hisG arg4::hisG/arg4::hisG sir2Δ::HIS1/sir2Δ::ARG4*
Bu_133	SN152. *URA3 heterozygous sir2Δ/Δ*	*MTL a/alpha ura3Δ-iro1Δ::imm434/URA3-IRO1 his1Δ/his1Δ arg4Δ/arg4Δ leu2Δ/leu2Δ sir2Δ::HIS1/sir2Δ::ARG4*
Bu_147	*TLO α10-URA3*$^{+}$ (3)	*ura3Δ::imm434 ura3Δ::imm434 his1::hisG/his1::hisG arg4::hisG/arg4::hisG TLOα10/ TLOα10-GFP-URA3-tADH*
Bu_148	*TLO α 12-URA3*$^{+}$ (3)	*ura3Δ::imm434/ura3Δ03::imm434 his1::hisg/his1::hisG arg4::hisG/arg4::hisG TLOα12/ TLOα12-GFP-URA3-tADH*
Bu_152	*TLO α 12-URA3*$^{+}$ *sir2Δ/Δ* (3)	*ura3Δ::imm434/ura3Δ03::imm434 his1::hisg/his1::hisG arg4::hisG/arg4::hisG TLOα12/ TLOα12-GFP-URA3-tADH sir2Δ::HIS1/sir2 Δ::ARG4*
Bu_153	*TLO α10-URA3*$^{+}$ *sir2Δ/Δ* (3)	*ura3Δ::imm434 ura3Δ::imm434 his1::hisG/his1::hisG arg4::hisG/arg4::hisG TLOα10/ TLOα10-GFP-URA3-tADH sir2Δ::HIS1/sir2 Δ::ARG4*
Bu_185	*rDNA-URA3*	*ura3Δ::imm434 ura3Δ::imm434 his1::hisG/his1::hisG ERG13/ERG13-GFP-URA3*
Bu_202	*rDNA-URA3 sir2Δ/Δ*	*ura3Δ::imm434 ura3Δ::imm434 his1::hisG/his1::hisG ERG13/ERG13-GFP-URA3 sir2Δ::HIS1/sir2 Δ::NAT*
Bu_234	*rDNA-URA3*$^{+}$ *csm1Δ/Δ*	*ura3Δ::imm434 ura3Δ::imm434 his1::hisG/his1::hisG ERG13/ERG13-GFP-URA3 csm1Δ::HIS1/csm1Δ::NAT*
Bu_253	*TLO γ16-URA3*$^{+}$ [3]	*ura3Δ::imm434 ura3Δ::imm434 his1::hisG/his1::hisG arg4::hisG/arg4::hisG TLO γ16/TLO γ16-GFP-URA3*
Bu_272	*Sir2-HA*	*MTLa/alpha tel::URA3 ura3Δ::Δimm434/ura3Δ::Δimm434 his1::hisG/his1::hisG arg4::hisG/arg4::hisG SIR2-HA*
Bu_277	*TLO γ16-URA3*$^{+}$ *sir2Δ/Δ*	*ura3Δ::imm434 ura3Δ::imm434 his1::hisG/his1::hisG arg4::hisG/arg4::hisG TLO γ16/TLO γ16-GFP-URA3 sir2Δ::HIS1/sir2Δ::NAT*
Bu_304	*NAT- TLO α10-URA3*$^{+}$	*ura3Δ::imm434 ura3Δ::imm434 his1::hisG/his1::hisG arg4::hisG/arg4::hisG VID21:NAT TLOα10/ TLOα10-GFP-URA3-tADH*
Bu_318	*TLO α10 ΔTRE-URA3*$^{+}$	*MTL a/alpha ura3Δ::Δimm434/ura3Δ::Δimm434 his1::hisG/his1::hisG arg4::hisG/arg4::hisG bts10Δ::URA3/bts10Δ::NAT*
Bu_323	*TLO α10 ΔTRE-URA3*$^{+}$ *sir2Δ/Δ*	*MTL a/alpha ura3Δ::Δimm434/ura3Δ::Δimm434 his1::hisG/his1::hisG arg4::hisG/arg4::hisG bts10Δ::URA3/bts10Δ::NAT sir2 Δ::HIS1/sir2 Δ::ARG4*
Bu_365	*rDNA:URA3 csm1Δ/Δ*	*rDNA::URA3 ura3Δ::λimm434/ura3Δimm434 his1::hisG/his1::hisG arg4::hisG/arg4::hisG csmΔ1::ARG4/csmΔ1::NAT*
Bu_450	SN152. *URA3 heterozygous csm1Δ/Δ*	*MTL a/alpha ura3Δ-iro1Δ::imm434/URA3-IRO1 his1Δ/his1Δ arg4Δ/arg4Δ leu2Δ/leu2Δ csm1Δ::NAT/csm1Δ::ARG4*

Supplementary table 2. Primers used in this study.

Primer	Sequence	Figure	Description
Bu_131	GACTGGCCAATTATAAATGTGAAGG	Fig5 A-B	Primer to check TRE deletion
Bu_139	GAGTGAGTGAGTGGAGTAGCG	Fig 1 D-E, Fig2, 4B, Fig5, FigS1B, FigS2D	Primer to check *sir2Δ/Δ* deletion
Bu_141	GTTGGGCAGATATTACCAATG	Fig5 A-B, FigS3, Fig3D	Primer to check URA3$^+$, TRE deletion
Bu_142	CCTTCACATTTATAATTGGCC	FigS3, Fig3D	Primer to check URA3$^+$
Bu_143	GACACTATATAGTGAGAATAAAATAATTCATTTGAAAAAAAATTGATAGTAAAAGATTTGGAGTAAATATTTTGGTTTTCCCAGTCACGACGTT	Fig1C-D	*URA3$^+$-rDNA*
Bu_144	GTAAAGCACATATGCATAATACTGGTCGAGAATTGAAAGATTGTTATTTTTTTCTAACAAAAAAAGAAACGCCATATGTGGAATTGTGAGCGGATA	Fig1C-D	*URA3$^+$-rDNA*
Bu_147	GTTCTAGCTTCTTCGCTAGTG	Fig1C-D	Primer to check *URA3$^+$-rDNA*
Bu_148	CAATTCGCCCTATAGTGAG	Fig1C-D	Primer to check *URA3$^+$-rDNA*
Bu_152	CTGGAGAAAATATAACCACGAGTCTAAGTTTCTTTATTATATTGACGTTTCAGTTATTTGAGAGAAATCCTCTAGTAGTTTTCCCAGTCACGACGTT	Fig 1 D-E, Fig2, 4B, Fig5, FigS1B, FigS2D	*sir2Δ/Δ* deletion mutant
Bu_153	ATATATAAATATATAAATATATATATATAAAAGAATTGAAAAGAAAAACATTAAAGACACCAATATTAATTTAATGTGGAATTGTGAGCGGATA	Fig 1 D-E, Fig2, 4B, Fig5, FigS1B, FigS2D	*sir2Δ/Δ* deletion mutant
Bu_164	CGGTCTGGTAAATGATTGAC	Fig1A,Fig1D, Fig2A,Fig4B,Fig5B,Fig5D,FigS1B, FigS2D	Primer to check *HIS1* integration
Bu_165	AGTGTGGAAAGAAGAGATGC	Fig1D-E,Fig 5 B, Fig2,FigS1B	Primer to check *ARG4* integration
Bu_179	CTGTATCTATAAGCAGTATCATCC	Fig1A, Fig1D,Fig2A-B, Fig4B, Fig5D,FIGS1B,Fi	Primer to check *NAT* integration

		g2SD	
Bu_274	CGTCGTTTTTTTTTTTCTTTTTGTTATATCCCCTTTAAACTATCAGCACATACACACCAACACCATTGTTTTCCCAGTCACGACGTT	Fig1A,Fig2, FigS1B	*csm1Δ/Δ* deletion mutant
Bu_275	CAAAAAACTTAAAGAAATTCTCAAGACATTGAAAATTACCAAAGTAGATGGCAAAATCGTTATAGAATAATAATGTGGAATTGTGAGCGGATA	Fig1A,Fig2, FigS1B	*csm1Δ/Δ* deletion mutant
Bu_276	GGGAGATCGAGACAATGGAT	Fig1A,Fig2, FigS1B	Primer to check *csm1Δ/Δ* deletion
Bu_286	CTGGAGAAAATATAACCACGAGTCTAAGTTTCTTTATTATATTGACGTTTCAGTTATTTGAGAGAAATCCTCTAGTAGTAAAACGACGGCCAGTGAATTC	Fig1A, Fig1D,Fig2A-B, Fig4B,Fig5B, Fig5D,FIGS1B,FiG2SD	*sir2Δ/Δ* deletion mutant
Bu_287	ATATATAAATATATAAATATATATATATAAAAGAATTGAAAAGAAAAACATTAAAGACACCAATATTAATTTAATGCATCAATTGACGTTGATACCAC	Fig1A, Fig1D,Fig2A-B, Fig4B,Fig5B, Fig5D,FIGS1B,FiG2SD	*sir2Δ/Δ* deletion mutant
Bu_288	CGTCGTTTTTTTTTTTCTTTTTGTTATATCCCCTTTAAACTATCAGCACATACACACCAACACCATTGTAAAACGACGGCCAGTGAATTC	Fig2A-B, FigS1B	*csm1Δ/Δ* deletion mutant
Bu_289	CAAAAAACTTAAAGAAATTCTCAAGACATTGAAAATTACCAAAGTAGATGGCAAAATCGTTATAGAATAATAATGCATCAATTGACGTTGATACCAC	Fig2A-B, FigS1B	*csm1Δ/Δ* deletion mutant
Bu_315	GATGAATTTAAATTATCAAAACTTAAAAATGGTGATTGGGAAATTGTCAAGAAATCAACTTCGACAAAAAAACGGATCCCCGGGTTAATTAA	FigS8B	*SIR2-HA tagging*
Bu_316	ATATATAAATATATATATATAAAAGAATTGAAAAGAAAAACATTAAAGACACCAATATTAATTTAATCAGTAAAACGACGGCCAGTGAATTC	FigS8B	*SIR2-HA tagging*
Bu_317	GATAGCCCGCATAGTCAGGAAC	FigS8B	Primer to check BTS HA tagged *SIR2-HA* mutant
Bu_356	GGCTTTGACATCAACGACA	Fig5 A-B	Primer to check TRE Δ deletion mutant
Bu_370	GCACATTTGGTGCCCACTCAAGCACTACACGCCTTGCTGTGTCCCCTGACCCTGCCGCAAGTGGAAGAGCCGTAAAACGACGGCCAGTGAATTC	Fig3E-F, FigS7A-B	*NAT- TLO α10-URA3*$^{+}$
Bu_371	CTAGCAACTCCTTTCCCAATAATTTCATTACCTCCTCGTCGGCAACATCTATTGTTTTACCTCTGGGATAATGCATCAATTGACGTTGATACCAC	Fig3E-F, FigS7A-B	*NAT- TLO α10-URA3*$^{+}$
Bu_372	CTAACCAATGTCAGACAACG	Fig3E-F, FigS7A-B	Primer to check *NAT-TLO α10-URA3*$^{+}$ construction

Bu_400	CGTAATCTGGAACGTCATATGG	Fig5 A-B	Primer to check TRE Δ deletion mutant
Bu_401	TACTGTTCCTGCAGAAGCCT	Fig5 A-B	Primer to check TRE Δ deletion mutant
Bu_402	TGCTCGACAATGGCGACCACGTAAACGAAGAGTTTGATGTAGACAGCTTTTTAAACCAGTTTGGTAATCCGCGGCGGATCCCCGGGTTAATTAA	Fig5 A-B	TREΔ with URA3/NAT
Bu_403	TTGAGTGGCGGGTCATTCTTTAGAGTGTTTGCAGGGTTGGTGGTGTTTGGCATTGGGTTGGCGTATGTGGTTCTAGAAGGACCACCTTTGATTG	Fig5 A-B	TREΔ with URA3
Bu_404	TTGAGTGGCGGGTCATTCTTTAGAGTGTTTGCAGGGTTGGTGGTGTTTGGCATTGGGTTGGCGTATGTGGTGTAAAACGACGGCCAGTGAATTC	Fig5 A-B	TREΔ with NAT
BU_606	TGCCCAAATGTCTTCCGAT	Fig6C-D	SNP1
BU_607	GAGGTAAGGGTTCAAGTCCA	Fig6C-D	SNP1
Bu_612	CTCGGGAGAATAAGCTTACCATCTG	Fig6A-B, Fig8	SNP85
Bu_613	TTACTTGTTGGGAAATCTGAACAGC	Fig6A-B, Fig8	SNP85

Supplementary table 3. Plasmids used.

**Plasmid**	**Description**
pGEMURA3	*URA3* integration products (4)
pGEMHIS1	*HIS1* substitution products (4)
pRS-Arg4SpeI	*Arg4* substitution products (4)
PHA_URA3	*HA-URA3* substitution products
pHA_NAT	*NAT* substitution products (5)

REFERENCE

1. Noble,S.M. and Johnson,A.D. (2005) Strains and Strategies for Large-Scale Gene Deletion Studies of the Diploid Human Fungal Pathogen Candida albicans. Eukaryotic Cell, **4,** 298.

2. Freire-Benéitez,V., Price,R.J., Tarrant,D., Berman,J. and Buscaino,A. (2016) Candida albicans repetitive elements display epigenetic diversity and plasticity. *Sci. Rep.*, **6**, 22989.

3. Anderson,M.Z., Gerstein,A.C., Wigen,L., Baller,J. a and Berman,J. (2014) Silencing Is Noisy: Population and Cell Level Noise in Telomere-Adjacent Genes Is Dependent on Telomere Position and Sir2. *PLoS Genet.*, **10**, e1004436.

4. Wilson,R.B., Davis,D. and Mitchell,A.P. (1999) Rapid Hypothesis Testing with Candida

albicans through Gene Disruption with Short Homology Regions These include : Rapid Hypothesis Testing with Candida albicans through Gene Disruption with Short Homology Regions. **181**.

5. Gerami-nejad,M., Forche,A., Mcclellan,M. and Berman,J. (2012) Analysis of protein function in clinical C . albicans isolates. **5314**.
